# Cell cycle-coupled transcriptional network orchestrates human B cell fate bifurcation

**DOI:** 10.1101/2025.04.23.649973

**Published:** 2025-06-25

**Authors:** Nicholas A Pease, Jingyu Fan, Swapnil Keshari, Jered Stratton, Peter Gerges, Betsy Ann Varghese, Narayanan VP Nampoothiri, Christopher S McGinnis, Wenxi Zhang, Steven B Gierlack, Tanush Swaminathan, Akanksha Sachan, Godhev Manakkat Vijay, Luis Mena Hernandez, Zarifeh Heidari Rarani, Camila Macedo, Diana Metes, Ansuman T Satpathy, Abhinav K Jain, Nidhi Sahni, Wayne Stallaert, Jishnu Das, Harinder Singh

**Affiliations:** 1 Center for Systems Immunology and Departments of Immunology and Computational and Systems Biology, University of Pittsburgh, Pittsburgh, PA; 2 The Joint CMU-Pitt Ph.D. Program in Computational Biology, Carnegie Mellon University and University of Pittsburgh, Pittsburgh, PA; 3 Deparment of Computational and Systems Biology, UPMC Hillman Cancer Center, University of Pittsburgh, PA; 4 Deparment of Pathology, Stanford University, Stanford, CA; 5 Parker Institute for Cancer Immunotherapy, San Fransico, CA; 6 Department of Surgery, University of Pittsburgh, Pittsburgh, PA; 7 Department of Epigenetics and Molecular Carcinogenesis, University of Texas MD Anderson Cancer Center, Houston, TX; 8 Quantitative and Computational Biosciences Program, Baylor College of Medicine, Houston, TX, USA

**Keywords:** Gene regulatory networks, human B cell differentiation, single-cell multi-omics, Map-Predict-Perturb, cell cycle-coupled cell fates

## Abstract

Antibody responses are determined by activated B cells bifurcating into plasmablasts (PBs) and germinal center B cells (GCBCs). Gene regulatory networks (GRNs) underlying human B cell fate choice remain uncharted. Temporally resolved single-cell multi-omics, computational modeling and CRISPR-based perturbations were used to assemble, simulate and test high-resolution GRNs underlying PB and GC fates. The results converged with orthogonal predictions of transcription factor (TF) action at single-nucleotide resolution, revealing dominant and reciprocal actions of IRF4 and its binding partners at simple and composite IRF motifs. Single-cell perturbation analysis of these TFs demonstrated multiple reciprocal negative feedback loops controlling the bifurcation. Additionally, IRF4 and BLIMP1, co-repressed the cell cycle regulators *MYC* and *CCND2*. G0/G1 lengthening accelerated the switching of cells to an IRF4^hi^BLIMP1^hi^ regulatory state and enhanced the probability of PB specification, thereby uncovering a self-reinforcing regulatory module that couples cell cycle dynamics to B cell fate choice.

## INTRODUCTION

Gene regulatory networks (GRNs) are central to the understanding of molecular mechanisms that orchestrate cell fate decisions and differentiation pathways^1^. While cell extrinsic factors promote differentiation, the divergence of multi- or bipotential progenitors into distinct specified cell states often occurs cell-autonomously and probabilistically - properties that are particularly manifested in tissue systems that do not have rigid spatial constraints such as compartments that enable immune cell development and elaboration of their varied effector states^2,3^. In keeping with these findings, though GRNs are modulated by cell-extrinsic signals, they can undergo reconfiguration in a cell-autonomous manner. The emergence and stable propagation of bifurcated cell states depend on cross-regulatory TF feedback loops within GRNs that emerge and evolve dynamically during differentiation^4,5^. Thus, to understand the genomic reprogramming which underlies the initiation and resolution of binary fate decisions, it’s critical to assemble and analyze high-resolution, temporally resolved GRNs. The expansion of single-cell analyses that simultaneously profile transcriptional and chromatin landscapes has facilitated the widespread application of GRN modeling across diverse developmental and cellular contexts^6,7^. However, GRN modeling is limited by drawbacks of individual computational genomic methods and sparsity of experimental testing. We sought to address both limitations.

B cells of the immune system represent an excellent and widely used model system to analyze bifurcating cell fate dynamics that underlie physiologically crucial antibody responses to infections or vaccines^8,9^. Generation of immediate and long-term humoral immunity requires naïve B cells to generate plasmablasts (PBs) and germinal center B cells (GCBCs) upon their activation. PBs are short-lived cells that secrete lower affinity antibodies, whereas GCBCs delay terminal differentiation to facilitate somatic hypermutation and affinity-based selection, ultimately potentiating the generation of memory B (MBCs) and long-lived plasma cells (PCs) that encode higher affinity antibodies^10^. We have previously demonstrated that the bifurcation of murine naïve B cells into PB and GC fates can be recapitulated *in vitro*^11^. An activation induced regulatory network involving sequential reciprocal negative feedback loops between the transcription factors (TFs) IRF4-IRF8 and BLIMP1-BCL6 has been proposed by us and others to orchestrate the generation of PB and GCBC states^11–13^. However, this GRN module has limited depth and range which is reflected in sparse TF nodes and their edges with *cis*-regulatory elements (CREs) of target genes. On the other hand, for human B cells, the EBV-transformed B cell line, GM12878, has been extensively profiled with a diverse set of genomic tools by many different groups and consortia^14^. However, the applicability of these genomic datasets and the underlying GRN to primary (non-transformed) PB and GCBC states and their emergent dynamics is limited. Therefore, we set out to assemble comprehensive state-specific GRNs underlying the bifurcating cell fate dynamics of primary human B cells and to couple their analysis with systematic *in silico* and experimental perturbations. Given the importance of both immediate as well as durable antibody responses to infections and vaccinations^9,15^, we attempted to systematically uncover and validate GRN modules that differentially control the human B cell fate bifurcation.

During their activation by cognate antigens, individual naïve B cell clones, in mice and humans, have been shown to give rise to both PB and GC fates, demonstrating that the cell fate choice is not necessarily pre-determined^9,16^. Additionally, clonal tracking of murine B cells by time-lapse imaging suggests PB differentiation occurs probabilistically over time^17,18^. However, the underlying molecular mechanisms that enable tuning of GC versus PB probabilities are not well understood. It is established that PB differentiation probability increases as a function of time and cell division number^19,20^. Nonetheless, the frequency of PB differentiation as a function of cell division is modifiable by intrinsic and extrinsic factors suggesting that cell division number *per se* does not determine cell fate^18,21,22^. Although probabilistic PB differentiation has been associated with cell cycle rates^18,23,24^, the underlying GRN modules that couple cell cycle dynamics to B cell fate choice have not been elucidated.

Herein, we assemble human B cell state-specific GRNs and in so doing, elucidate a cell cycle-coupled transcriptional module that governs the bifurcation of activated cells into PB and GC fates. Single-cell transcriptional profiling and clonal tracking demonstrates that human B cell fate bifurcation can be recapitulated *in vitro*, with PB and GC fates determined probabilistically following cell division. Temporally-resolved single-cell multi-omics along with complementary computational genomic tools -each overcoming the limitations of individual methods- are used to construct the first high-resolution, human B cell state-specific GRNs. The quality of the assembled GRNs was validated by systematic simulated perturbations of central TFs within B cell GRNs followed by testing of their impact using CRISPR editing. We provide a UCSC genome browser session (https://tinyurl.com/BcellMultiomeTracks) and a user-friendly web application resource (https://pitt-csi.shinyapps.io/humanBcellGRN/) that will facilitate analyses of human B cell states and predicting their dynamics during pathogen and vaccine responses as well as analysis of non-coding variants impacting B cell-mediated autoimmune diseases. Strikingly, the GRN analysis converged with predictions of chromatin accessibility contribution models that inferred state-specific action of TFs at single-nucleotide resolution within all catalogued *cis*-regulatory elements (CREs) revealing reciprocal action of IRF4 and its interaction partners at IRF composite elements and interferon sequence response elements (ISREs) in GCBC and PB states, respectively. Single cell perturbation analysis of these GCBC-inducing and PB-promoting TFs uncovered multiple reciprocal negative feedback loops controlling the bifurcation. Additionally, IRF4 and BLIMP1, co-repressed the cell cycle regulators *MYC* and *CCND2*. G0/G1 lengthening accelerated the switching of cells to an IRF4^hi^BLIMP1^hi^ regulatory state and enhanced the probability of PB specification, thereby uncovering a self-reinforcing regulatory module that couples cell cycle dynamics to B cell fate choice.

## RESULTS

### Human B cells activated *in vitro* bifurcate into PB and GC fates

**Figure 1.**
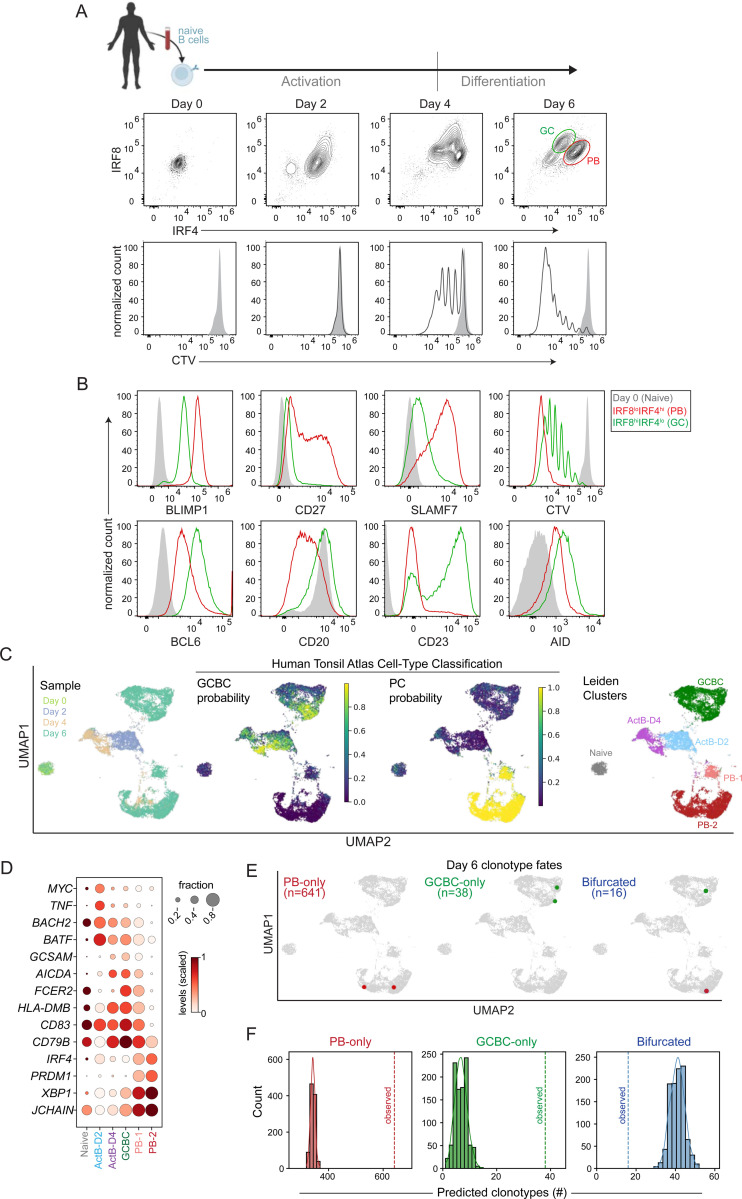
Human B cells activated *in vitro* bifurcate into PB and GC fates. (A) Naïve B cells, isolated from healthy human donor PBMCs, were labeled with CellTrace Violet (CTV) and stimulated *in vitro* as indicated in the schematic (*top*) (see Methods). Inducible expression of the transcription factors, IRF4 and IRF8, was monitored by intracellular flow cytometry on indicated days (*middle*). Cell proliferation was simultaneously monitored by measuring CTV dilution at each timepoint (*bottom*). (B) Flow cytometry analysis of differentiated human B cells for indicated proteins and CTV based on PB and GC gates (D6, Figure 1A). (C) Single-cell RNA-seq/BCR-seq profiling of *in vitro* activated B cells at D0, D2, D4, and D6. Cells in the aggregated UMAP are colored by sample timepoints (*left*), support vector machine classification probabilities trained on Human Tonsil Atlas B cell states (*middle*), and Leiden cluster annotations (*right*). (D) Expression of representative marker genes in annotated Leiden clusters. Dot size represents the fraction of cells expressing the indicated transcripts and color intensity indicates the mean expression level, standardized between 0 and 1. (E) UMAP display of representative B cell clonotypes based on D6 fates. The number (n) of clonotypes in each cell fate category (PB-only, GC-only and bifurcated) are indicated in the panels. (F) Expected versus observed number of unique clonotypes (clone size >= 2) per indicated cell fate category. Distribution of expected clonotype counts were derived using Monte Carlo simulations based on the Poisson distribution of observed clonotype sizes in Figure S1F. All analyses were performed with B cells from Donor 1 (male).

Previous *in vitro* studies of human B cell activation and differentiation have primarily focused on the generation of PBs and not analyzed the alternative GC fate^25–27^. To enable robust generation and genomic analyses of bifurcating PB and GC trajectories, we adapted an existing *in vitro* differentiation system. This involved signaling-induced activation of naïve B cells with anti-IgM, CD40L, IL-2 and CpG (4 days) that promoted proliferative bursting. This was followed by re-culturing of cells with IL-10 and IL-4 while retaining CD40L and IL-2 (2 days) to enable optimal GCBC and PB differentiation^25^ (Figure 1A). In contrast with previous studies^25,26^, we chose to retain CD40L in the culture system during the differentiation phase because it mimicked sustained T cell help and increased the frequency GC cells (see below). We chose to analyze the differentiation of human B cells based on the bifurcated expression patterns of the activation-induced TFs, IRF4 and IRF8, which are observed in the murine system and also in human HA-specific B cells that emerge within one week of influenza immunization^8,11^. Within 24h, a fraction of the activated B cells had entered S phase (Figure S1A). At two days post-activation, most of the B cells uniformly induced expression of IRF4 and had upregulated IRF8 (Figure 1A). At this stage some of the cells had undertaken their first cell division (Figure S1A). After four days of activation, the activated B cells began to manifest bifurcated expression of IRF4 and IRF8 and had undergone 1–4 cell divisions (Figure 1A). Upon transfer into media containing IL-4 and IL-10 while retaining CD40L and IL-2, the B cells bifurcated into distinctive IRF8^lo^IRF4^hi^ and IRF8^hi^IRF4^lo^ regulatory states. Notably, the generation of these bifurcated B cell states was reproducible across 8 different donors (4 female and 4 male) of different ethnicities and ages (Figure S1B).

To characterize the differentiated B cell states (D6) associated with the bifurcated expression of IRF4 and IRF8, we analyzed the levels of the counteracting PB and GCBC TFs, BLIMP1 and BCL6 as well as various PB and GCBC markers. As expected, the IRF8^lo^IRF4^hi^ cells exhibited the hallmarks of PBs with higher expression of BLIMP1, CD27 and SLAMF7 and low expression of BCL6, CD20, CD23 and AID (Figure 1B). In contrast, the IRF8^hi^IRF4^lo^ cells exhibited higher levels of the pro-GC TF, BCL6, as well as CD23 and AID, which are highly expressed in light-zone and dark-zone GCBCs of human tonsil tissues, respectively^28^. Consistent with CD40 signaling-dependent GCBC formation *in vivo*^29^, the frequency of the *in vitro* generated GCBC-like cells increased as a function of concentration of CD40L during the differentiation phase (Figures S1C and S1D). Proliferation dye (CellTrace Violet, CTV) labeling analysis revealed proliferation during both stimulation steps with the cells undergoing 2–3 cell divisions between D4 and D6 (Figure 1A). Notably, the PB fate represented progeny of cells that had undergone more extensive cell divisions than the GC fate (Figure 1B). Together, these results suggest that the *in vitro* system can robustly recapitulate the bifurcation of activated human B cells into PB and GC fates.

To enable high dimensional analyses of the divergent PB and GC fates and substantiate the correspondence of their transcriptomic states with their *in vivo* cellular counterparts in human tonsillar tissue, we performed single-cell RNA-sequencing (scRNA-seq). The resulting gene expression matrices of individual cells, generated by temporal profiling (D0, D2, D4, and D6) were analyzed by MIRA-based probabilistic topic modeling^30^. In this framework, each cell was represented by a combination of latent topics, where each topic is defined by a specific set of genes that can jointly reflect an underlying biological pathway or process. The framework reduces the complexity of high-dimensional yet sparse scRNA-seq data into interpretable gene programs, to enable ordering of diverse cellular states. Thus, each cell was modeled as a mixture of weighted gene programs, enhancing the detection of transcriptional differences between cell states. We projected the topic representations of all cells (D0–6) in two-dimensions using uniform manifold approximation projection (UMAP) (Figure 1C). D2 cells showed a substantial alteration in their transcriptional state in relation to naïve cells, while not manifesting a bifurcation. In contrast, a fraction of D4 cells were seen to diverge into two major transcriptional states, congruent with their bifurcating IRF4 and IRF8 levels (Figure 1A). As expected from the phenotypic analysis, the D6 cells were distributed among two major transcriptional states. We used Support Vector Machine (SVM) learning for unbiased classification of cells in our dataset with their *in vivo* counterparts. Specifically, we used the scRNA-seq datasets from the Human Tonsil Atlas^31^ to train a SVM model focusing on the major B cell states annotated in the atlas: naïve, activated B cell (ActB), memory B cell (MBC), GCBC, and PC. This analysis supported the conclusion that the major B cell states generated *in vitro*, between D4-D6, corresponded to their *in vivo* GCBC and PB/PC counterparts (Figure 1C).

Leiden clustering of cells from all timepoints enabled their resolution, based on topic modeling, into discrete transcriptional states that were annotated as Naïve, ActB-D2, ActB-D4, GCBC and PB-1,2 (Figure 1C, right panel; see Table S1 for marker gene analysis). Activated B cell signature genes, *TNF* and *MYC*, were transiently up-regulated in the ActB-D2 state while GC-promoting transcription factors, *BACH2* and *BATF*, were induced in the ActB-2 state and persisted through the GCBC state (Figure 1D). Notably, the GCBC cluster exhibited the highest levels of GC-associated genes including *GCSAM*, *AICDA*, *FCER2*, *HLA-DMB*, *CD83* and *CD79B.* A subset of cells in this cluster displayed high MYC-target gene as well as G1/S and G2/M scores (Figure S1E). In contrast, the two PB clusters, corresponding to distinct maturation states, exhibited the highest levels of the PB regulators, *IRF4* and *PRDM1* (BLIMP1), as well as genes associated with antibody secretion including *XBP1* and *JCHAIN*. These results showed that the bifurcated transcriptional states of PBs and GCBCs (D6) resemble their *in vivo* counterparts (Figures 1C and 1D; Table S1) and appear to diverge from those manifested by bipotent ActB precursors.

We next sought to determine whether B cell fate choice is pre-determined or emerges stochastically after activation and cell division. To do so, we tracked the fates of individual B cell clones by using paired single-cell RNA and B cell receptor (BCR)-sequencing data. Among the identifiable clonotypes containing at least 2 cells within the D6 sample (n=695), 641 were comprised of only PBs, 38 of only GCBCs and 16 were a mix of PBs and GCs (Figures 1E and 1F). The overrepresentation of PB-only clones in the D6 sampling was to be expected given that the PB fate was associated with greater number of cell divisions than the GCBC fate (>7 vs. 1–6 cell divisions, Figure 1B). We used the Poisson distributions of the observed PB and GC clone size frequencies (Figure S1F) to test the null hypothesis that the two fates manifest independently of one another, regardless of intra-clonal relationships. The observed number of both PB-only and GC-only clonotypes significantly exceeded the expected number in each category, under the assumption that clonal sibling fates are completely independent (n=336 PB-only (p ≈ 0) and n=7 GC-only (p < 10e-10)) (Figure 1F). The data were therefore consistent with a model in which, following probabilistic cell fate specification (PB or GC), the differentiated cell states are stably maintained in clonal progeny. In agreement with this model, the observed number of bifurcated clonotypes (n=16) was lower than expected (n=38, p=5e-9). Nonetheless, the presence of bifurcated clonotypes suggested that the fates of individual naïve B cells are not pre-determined but emerge independently across and within clonal families. Therefore, these results, in conjunction with the initial manifestation of divergent transcriptional states at D4 (Figure 1C), are most consistent with a model in which activated B cells probabilistically specify into PB or GC fates after activation and cell division between D2 and D4.

### Assembly of GRNs underlying divergent human B cell fate trajectories

**Figure 2.**
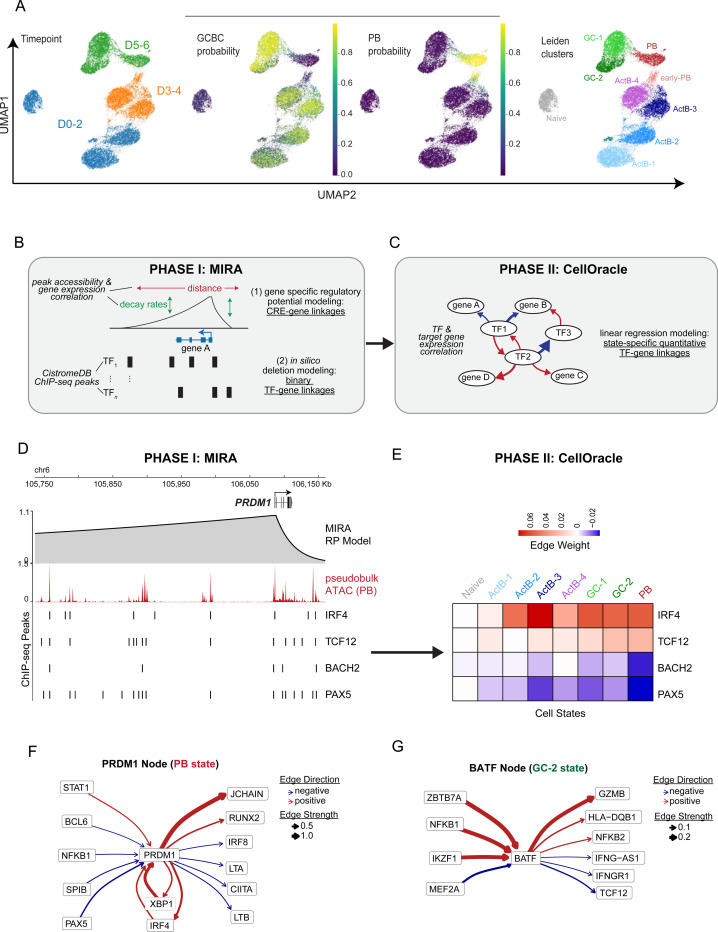
Assembly of GRNs underlying bifurcation of human B cells into PB and GC fates. (A) Naïve human B cells from Donor 1 (male) were activated and differentiated, *in vitro*, as in Figure 1A and profiled every 24 hours by paired single-cell RNA/ATAC-seq. MIRA joint topic modeling of transcripts and chromatin accessibility features^30^ was used to project cells in low-dimensional UMAP space and colored based on timepoint (*left*), support vector machine classification probabilities based on Human Tonsil Atlas cell-types (*middle*), and Leiden clusters with their cell-type annotations (*right*). (B) Schematic for assembling qualitative TF-gene linkages using MIRA-based RP and ISD modeling^30^. Gene-specific regulatory potential (RP) models are generated from learned decay rates and distances based on the correlation between peak accessibility and gene expression values derived from multiome datasets. Regulatory potential of TFs was inferred by measuring the change in each RP model following probabilistic in silico deletion (pISD) of TF binding regions (CistromeDB ChIP-seq peaks). (C) Schematic for delineating quantitative and directional TF-gene linkages using CellOracle GRN modeling. Cell state specific linear regression models were generated for each TF-gene pair derived from MIRA in Phase I. (D) Representative RP model for *PRDM1* gene. Each gene-specific model predicts the regulatory potential of ATAC-seq peaks flanking the gene transcription start site. ATAC-seq signals derived from the PB state pseudobulk are shown below the RP model. ChIP-seq peaks (CistromeDB) that overlap with ATAC-seq peaks for representative TF regulators predicted by MIRA pISD modeling are shown below. (E) Representative CellOracle state-specific TF-gene edge weights for MIRA predicted regulators of *PRDM1*. (F-G) Representative sub-GRNs derived from CellOracle GRN modeling. Select incoming and outgoing edges for the PRDM1 node in the PB state or the BATF node in the GC-2 state are displayed with thickness of arrows corresponding to state-specific edge weight values. Red and blue edges indicate positive and negative regulation, respectively.

To assemble high-resolution GRNs that underly the bifurcating B cell fate trajectories, we performed temporal single-cell multi-omics, jointly profiling gene expression and chromatin accessibility at 24h intervals. For these analyses, we used two different donors (male and female). To resolve discrete B cell states, we performed MIRA-based joint topic modeling, representing each cell according to weighted mixtures of gene expression and chromatin accessibility topics^30^ (Figures 2A and S2A). These expression topic gene sets were enriched for a wide-range important immune-cell pathways (Figure S2B, Tables S2 and S3), while the accessibility topic peak sets were enriched for diverse sets of TF binding regions including those for known regulators of murine PBs and GCBCs, based on over ten thousand ChIP-seq datasets from the Cistrome Data Browser (CistromeDB) (Figure S2B, Table S4). As with the scRNA-seq dataset (Figure 1C), SVM training on *in vivo* B cell states derived from the Human Tonsil Atlas^31^ was used to classify each cell in our multiome datasets (Figure 2A, *middle panels*). Cells from the D0-D2 timepoints were predominately classified as naïve or ActB states (data not shown), whereas the probability of GCBC and PB classification increased at D3–4 timepoints as the ActB probability decreased. Finally, in agreement with the scRNA-seq analysis (Figure 1C), D5-D6 cells were predominately classified as GCBC or PB. Leiden clustering identified eight B cell states annotated as naïve, ActB-1 to ActB-4 (corresponding to D1 to D4), GC-1, GC-2 and PB (Figures 2A and S2A, *right panels*).

To comprehensively assemble human B cell state-specific GRN models composed of inferred TF-gene linkages, we coupled two complementary GRN modeling frameworks that have previously been used independently of one another. The following considerations motivated our integrated computational design. GRN models are traditionally based on inferring TF-gene linkages by assessing TF motif enrichment at candidate cis-regulatory elements (cCREs) that are dynamically co-accessible with the promoters of genes using the Cicero genomics tool^32,33^. This approach has two limitations: (1) genes can be dynamically regulated independently of alterations in cCREs and promoter accessibility and (2) a given TF motif can be bound by different structurally related or even unrelated TFs. To overcome these two limitations, the first phase of our GRN assembly utilized MIRA regulatory potential (RP) modeling to establish binary TFgene linkages (Figure 2B). Instead of relying on promoter-and-cCRE accessibility correlations, MIRA takes advantage of single-cell multiome data to construct RP models for each dynamically expressed gene based on the correlation between accessibility of associated cCREs and transcripts of the gene. In RP modeling, the influence of a cCRE on a gene’s transcription decays exponentially with genomic distance from the transcription‑start site (TSS), an assumption supported by chromatin‑conformation data such as Hi‑C^34^. In the RP models the impact of cCREs on gene activity are additive. Additionally, MIRA leverages over ten thousand uniformly processed ChIP-seq datasets in CistromeDB rather than TF motif enrichment to infer binding of annotated TFs to cCREs. Probabilistic *in silico* deletion of the TF binding regions are then used to generate gene specific models which predict TFs that likely regulate a given gene based on the change in the model’s ability to predict transcript levels from dynamic accessibility features when TF binding peaks overlapping with these regions are removed. This approach has successfully been applied to robustly predict TF-gene linkages^35,36^. Thus, in Phase I, the MIRA assembled GRN is based on dynamic chromatin and gene expression profiles of human B cells and their integration with evidence of physical TF-cCREs interactions based on CistromeDB ChIP-seq datasets, providing a robust set of TF-gene linkages for downstream network refinement.

**Figure 3.**
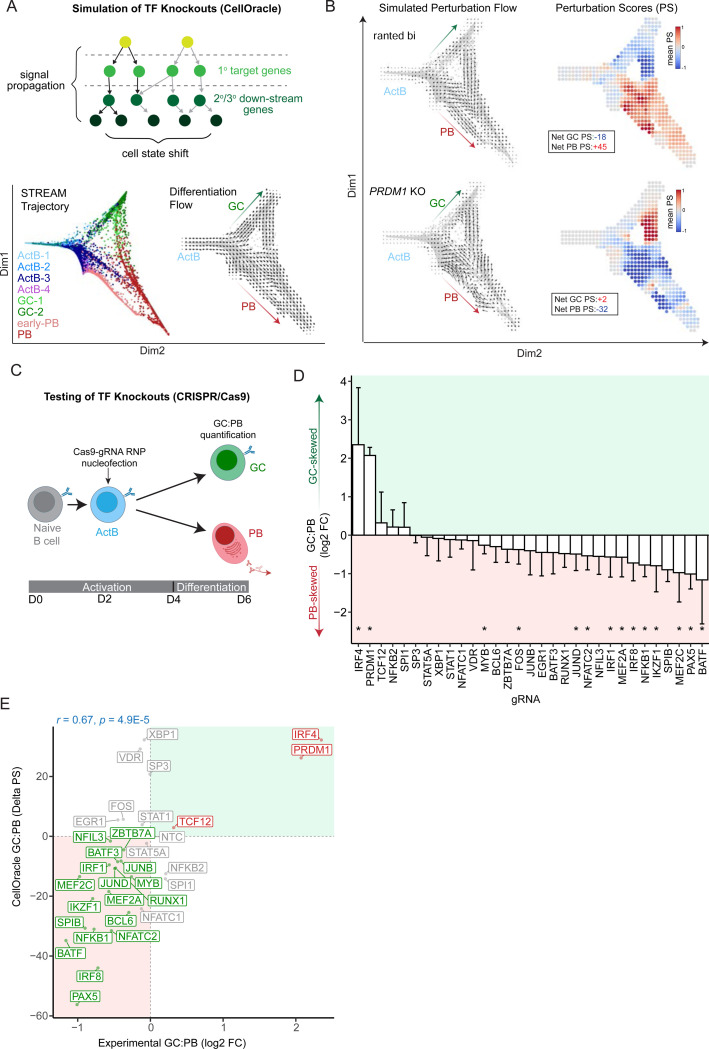
Predicting and testing TF control of human B cell fate choice. (A) Schematic depiction of *in silico* TF knockouts (KO) via signal propagation in CellOracle GRN models (*top*). Cells from Donor 1 multiome data were projected in low-dimensional space according to STREAM-inferred trajectory analysis (*bottom left*). STREAM-pseudotime values were used to infer the differentiation flow in the CellOracle gradient module and displayed as a vector field graph (*bottom right*, see Methods for details). (B) Representative *in silico* TF KO simulations visualized with perturbation flow vector fields (*left*) or perturbation score grids (*right*) for PAX5 and PRDM1 (Donor 1). Perturbation scores (PS) are the dot product of differentiation flow vectors (Figure 3A, *bottom right*) and perturbation simulation vectors. Net GC and net PB perturbation scores (PS) are derived from summing the PS values for cells in GC or PB states, respectively. (C) Experimental strategy for testing the predicted effects of CellOracle based TF KO simulations on B cell fate choice. Naïve B cells were nucleofected with RNP complexes targeting individual TF genes at D2 post-activation and cultured for four days as in Figure 1A before quantifying PB and GCBC cells by flow cytometry (see Figure S3C for gating strategy). (D) Experimental results of arrayed CRISPR TF KO screen. Fold change of GC:PB ratio (D6) of KO vs. NTC (non-targeting control) are displayed for indicated TFs (n = 4, two experimental replicates each for Donor 1and Donor 2 and n = 2 for SPIB for Donor 1), *q-value < 0.05). (E) Comparison of simulated and experimental TF KO effects on B cell fates. Delta PS values (Net GC PS - Net PB PS, see Figure 3B) for indicated simulated TF perturbations are plotted with their experimentally determined log2-FC in GC/PB ratios measured by CRISPR perturbations and flow cytometry analysis (R = Spearman’s rank correlation coefficient). TFs with concordant predicted and experimental impact on B cell fate determination (>= 25% change in GC:PB ratio) are displayed in red (PB-promoting TF) or green (GC-promoting TF).

In the second phase of GRN assembly (Figure 2C), we use the binary TF-gene linkages derived from MIRA as a base-GRN that serves as the input for the inference of context-specific GRNs using CellOracle^33^. As we experimentally test and demonstrate later, this base-GRN is of significantly higher quality than the default base-GRN of CellOracle derived using Cicero. The inference of cell-state-specific GRNs utilizes cell state-specific Bayesian ridge regression models that fit the transcript levels of each TF and its predicted target gene (measure of TF contribution to gene activity) for each cell cluster. This enables TF-gene linkages to be assigned quantitative contribution values (strengths) as well as signs (activation or repression). These transformed TF-gene linkages can then be used to infer the centrality of each TF to a given cell state. A methodological advance is the coupling of MIRA and CellOracle that significantly enhances the quality of the base-GRN and makes the false discovery rate (FDR) control of inferred regulatory relationships more stringent (see testing in Figures 3 and 5).

To illustrate Phase I, we show the MIRA RP models for the PB-associated gene *PRDM1* (Figure 2D) and the GCBC-associated gene *AICDA* (Figure S2C) as exemplars. The relative importance of each cCRE is weighted as a function of bi-directional learned decay rates and distances from the gene’s TSS. Notably, the gene-specific RP models generally captured the TSS-cCRE interactions that were detected by chromatin conformation capture assays of accessible regions (HiCAR) in the GM12878 human B cell line^37^ (see UCSC Genome Browser session: https://tinyurl.com/BcellMultiomeTracks). The cCREs in these gene-specific RP models were annotated with CistromeDB ChIP-seq TF binding datasets (selected TF ChIP-seq peaks are displayed below ATAC-seq peaks in Figures 2D and S2C). This in turn enabled prediction of the regulatory impact of a given TF on its target gene by probabilistic *in silico* deletion (ISD) analysis (see Methods). The top 5% of TF-gene ISD scores were then used to generate binary TF-gene linkages that served as the base-GRN for inference of context-specific GRNs using CellOracle.

In Phase II, CellOracle transformed the binary TF-gene linkage predictions of MIRA into quantitative TF-gene edge strengths for each B cell state (Table S5). For example, CellOracle predicted IRF4 and TCF12 as positive regulators of the *PRDM1* gene and PAX5 and BACH2 as negative regulators, each with dynamic edge strengths across the various B cell states (Figure 2E). In contrast, CellOracle predicted PAX5, IRF4 and BATF, as positive regulators of *AICDA*, in the ActB and GC states (Figure S2C). We note that IRF4 has been shown to regulate to positively regulate both *AICDA and PRDM1* gene expression in activated and differentiating murine B cells^13^ and suggested to do so in a concentration and context-dependent manner. Accordingly, the dynamic GRN linkages in our state-specific models infer IRF4 as a positive regulator of *AICDA* in the ActB and GC states but as a repressor in the PB state.

To systematically examine the relative importance of TFs within each state-specific GRN we retained the top 10,000 statistically significant TF-gene edges (p < 0.001) and, for each cell state, calculated each TF’s eigenvector centrality, which is proportional to the influence of all its connected nodes. To examine how the relative centrality of each TF changes in each of the B cell states, we scaled the TF eigenvector centrality scores across cell states and performed k-means clustering which revealed six primary groups of TF nodes (Figure S2D, Table S6). The first group which was most central to the naïve B cell GRN, contained FOXO1, KLF4 and KLF6 which function in B cell development^38,39^. Reconfigured GRNs in ActB-1 and ActB-2 states were dominated by signaling-induced TFs including BATF and NFKB1. Subsequently, the next major wave of TF activity emerged in ActB-3 and ActB-4 states with heightened centrality of BCL6, EGR1 and SPIB, followed by NR4A3, MYB, MEF2A, POU2F1, SPI1, and NFKB2 in the GC-1 and GC-2 states, respectively. In contrast, the PB GRN was dominated by POU2AF1, PRDM1, CREB3L2, XBP1, and IRF4. To illustrate regulatory linkages that serve as inputs into a central TF node as well as its outputs, we illustrate the *PRDM1* and *BATF* nodes in the PB and GC-2 states, respectively (Figures 2F and 2G). In addition to quantitatively predicting repressive inputs into *PRDM1* by TFs that promote GCBC differentiation namely, BCL6, NFKB1 and SPIB the *PRDM1* PB node included reciprocal positive feedback with two other core PB TFs, IRF4 *and* XBP1^40^. In contrast, the BATF GC-2 node included positive incoming regulatory edges from GC-associated TFs, including ZBTB7A, NFKB1, and IRF4, and positive outgoing regulatory edges to genes associated with B cell activation (*HLA-DQB1, NFKB2*)^41^. Thus, the integrated MIRA-Cell Oracle framework, utilizing dynamic single cell chromatin accessibility and gene expression features, enabled assembly of high-resolution state-specific GRNs for activated and differentiated human B cells. The integrated analysis provides a catalogue of predicted human B cell state-specific TF-gene linkages via cCREs and inferred dynamic human TF networks that undergo pronounced reconfigurations during B cell activation, and the specification of alternate GCBC and PB cell fates. To facilitate use of the datasets and the GRN predictions, we provide a UCSC Genome Browser session (https://tinyurl.com/BcellMultiomeTracks), which includes state-specific ATAC-seq (cCRE) profiles and gene-specific RP models, along with a user-friendly web application resource (https://pitt-csi.shinyapps.io/humanBcellGRN/).

### Predicting and testing TF control of human B cell fate choice

The assembly of human B cell state-specific GRN models comprised of quantitative TF-gene linkages, enabled comprehensive testing of the impact of individual TFs on GCBC versus PB cell fate determination by simulating their perturbations *in silico*. CellOracle assessed such perturbations by propagating loss of expression of a given TF across the inferred GRN via its primary, secondary and tertiary target genes (Figure 3A). The resulting changes in gene expression values were used to estimate shifts in cell states. We performed the perturbation modeling with 32 different TF genes that displayed a range of GRN centrality scores (Donor 1 and Donor 2) and were therefore expected to differentially impact the GC and PB cell fate bifurcation (Figure S2D, Table S6). To represent cells along the bifurcated trajectories, we applied the STREAM pipeline, which infers differentiation branch points and pseudotime values based on variable gene expression patterns^42^ (Figure 3A, left panel). These pseudotime values were then used to generate a differentiation gradient which estimates the normal flow of differentiation represented by a vector field graph with CellOracle (Figure 3A, right panel). The impact of perturbations of the counteracting TFs PAX5 and PRDM1, within the male and female donor B cell GRNs are shown (Figure 3B and S3A). Alterations in the differentiation flow were visualized with perturbation flow vectors projected onto the STREAM trajectories. Perturbation scores for GC and PB cell states were calculated based on the dot product of differentiation flow vectors and perturbation flow vectors. The simulations correctly predicted the expected PB- and GC-skewing impact of loss of PAX5 and PRDM1, respectively^12^. The TF knockout (KO) simulations produced a wide range of predicted effects on the GC:PB ratio which were highly concordant between the male and female GRN models (Figure S3B). For example, both models predicted strong negative effects on the GC:PB ratio, i.e., decreased GCBC formation for the known GC-associated TFs PAX5, IRF8, SPIB, and BCL6^43^. Conversely, both models predicted strong positive effects on the GC:PB ratio, i.e., increased GCBC formation for KOs of PB-associated TFs such as XBP1, PRDM1 and IRF4.

To test these *in silico* predictions, we used an arrayed CRISPR screen to KO the corresponding TF encoding genes and assay their impact on the GC:PB ratio using the *in vitro* system. Naïve B cells for each donor were activated for two days before nucleofection with RNP complexes containing gRNAs targeting a given TF gene (Figure 3C). The cells were then cultured for four additional days as detailed in Figure 1. GCBC and PB cell numbers were quantitated by flow cytometry (D6). GCBCs included cells which were CD20^hi^CD27^lo^ and additionally expressed high levels of either CD23 and AID, markers of GCBC states^28^ (Figure S3C). PBs were enumerated by gating on cells that were CD27^hi^CD38^hi^SLAMF7^hi^. CRISPR editing efficiency was confirmed by extracting DNA from cells on Day 4 for PCR genotyping (Figure S3D). The results of the experimental screen, integrated for the male and female donor, revealed 12 pro-GC TFs with statistically significant impact that resulted in reduced GC:PB ratios including PAX5, BATF, IRF8 and NFKB1. In contrast, the screen identified only two pro-PB TFs, namely IRF4 and PRDM1, whose loss resulted in a significant increase in the GC:PB ratio (Figure 3D). Importantly, the simulated and experimental GC:PB ratios resulting from the TF perturbations were highly correlated (Spearman’s rank correlation r = 0.67, p = 4.9e-6) (Figure 3E). This correlation was substantially better than one generated by comparing the experimental results with simulations performed using the default Cicero base-GRN input into CellOracle (r = 0.48, p = 6e-3). Given the improved performance, the results testified to the importance of coupling MIRA and CellOracle models for assembling state-specific GRNs and simulating TF perturbations. Furthermore, the large effect sizes of the IRF4 or PRDM1 perturbations suggested their coordinated action in specifying the PB fate while antagonizing the GCBC fate.

### ChromBPNet uncovers dominant and reciprocal TF action at IRF motifs during B cell fate specification

**Figure 4.**
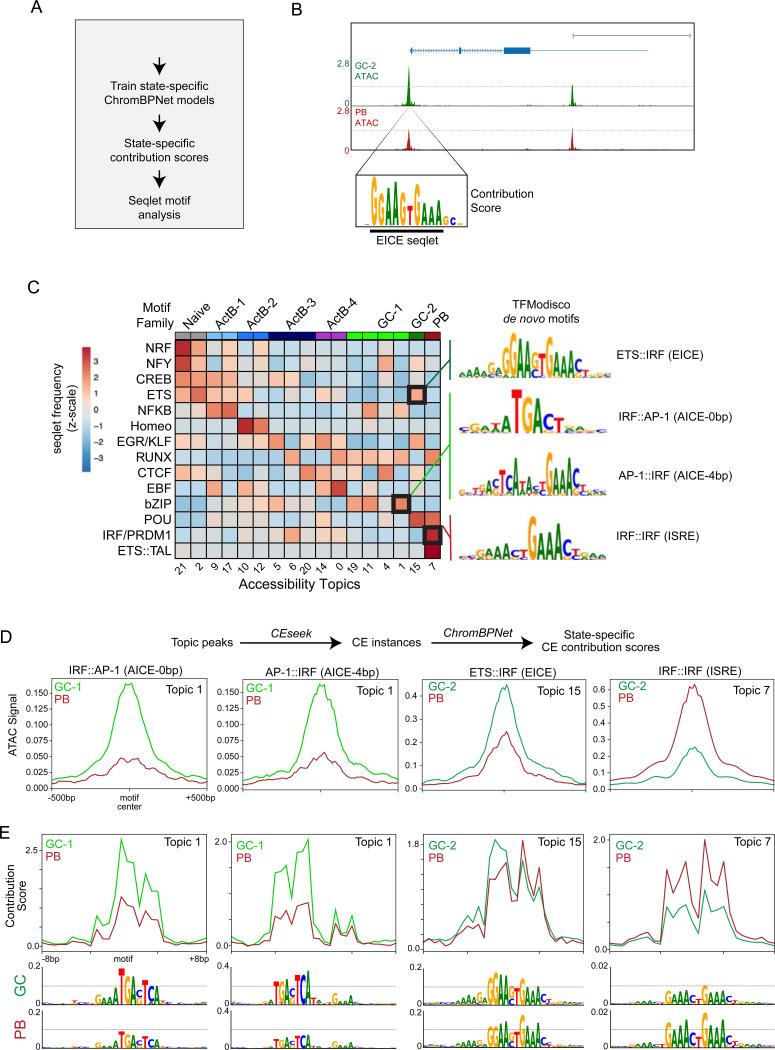
ChromBPNet uncovers dominant and reciprocal TF action at IRF motifs during B cell fate specification. (A) Analytical scheme for cell state-specific ChromBPNet modeling and analysis. (B) UCSC genome browser view of representative locus with ATAC-seq tracks in GC-2 and PB states along with highlighted ChromBPNet contribution score signals with annotated TF motif seqlets at a representative ATAC-seq peak. (C) Heatmap displaying the TFModisco-derived seqlet frequencies (z-scaled across chromatin accessibility topic columns) for a given TF motif family. Analysis was performed using top 10,000 peaks of indicated state-specific accessibility topics paired with their state-specific ChromBPNet models. Seqlets were annotated using Jaspar 2024 motif cluster families and z-scaled across columns reflective of indicated cell states. Only motif families with a seqlet frequency of at least 5% within at least one topic are displayed (*left*). CWMs for dominant IRF-motif containing elements in GC (EICE and AICE) vs PB (ISRE) states are shown (*right*). (D-E) Analysis of GC- or PB-associated topic peaks containing IRF-related composite element sequences by combining CEseek composite element motif scanning with ChromBPNet. (D) Aggregated ATAC-seq signals are displayed for GC or PB cell states at indicated topic peaks containing of IRF-related composite elements (EICE, AICE) or motifs (ISRE). (E) Aggregated contribution score signals (*top*) and CWM logos (*bottom*) are displayed for GC or PB cell states at indicated topic peaks containing of IRF-related composite elements (EICE, AICE) or motifs (ISRE).

The simulated and experimental TF perturbation screens demonstrated that IRF4 and BLIMP1 are the most dominant regulators of PB differentiation. However, IRF4 is also uniquely important for murine GCBC generation. It has been suggested that this dual role of IRF4 in PB fate specification and GC formation is determined by graded changes in IRF4 protein levels^13^. IRF4 has been shown to interact with different partner TFs and recognize distinct composite elements (CEs) within CREs. Notably, three of the GCBC-associated TFs (IRF8, SPIB, BATF) identified in our simulation and experimental screens bind with or replace IRF4 at ETS-IRF CEs (EICE); AP1-IRF CEs (AICE) and ISREs^13,44,45^. In contrast, the other dominant PB-inducing TF, BLIMP1, binds to a motif that is nearly identical to the ISRE and has also been shown to compete with PU.1/SPIB for binding at EICE motifs in activated murine B cells^46^. These considerations raised the possibility that graded expression of IRF4, in concert with dynamic changes in the levels of its partner TFs, dictate the probabilities of GC and PB fate specification. To explore this hypothesis in an unbiased manner we utilized ChromBPNet modeling. ChromBPNet is a convolutional neural network (CNN) that learns the contribution of individual base-pairs to the accessibility of ATAC-seq peaks within which they are embedded, after regressing out Tn5 enzyme cutting bias^47,48^. In so doing it learns the TF motif lexicon and infers dynamic TF action at CREs. We applied this CNN framework to our multiome dataset by training state-specific ChromBPNet models on pseudobulk ATAC-seq reads from each cell cluster. Each of these state-specific models was then used to generate corresponding base-pair resolution contribution score (CS) predictions across the union set of ATAC-seq peaks (Figure 4A). These state-specific contribution score tracks revealed short DNA sequences, referred to as “seqlets”, that were predicted to be important in determining the accessibility profiles of their corresponding ATAC-seq peaks. Strikingly, many of the ChromBPNet seqlets corresponded to known TF motifs thereby implicating action of cognate TFs in binding to such sequences and controlling chromatin accessibility. An example of a seqlet (EICE motif) contributing to the accessibility of a CRE at the *TLR4* locus in the GC-2 cell state is shown in Figure 4B.

To evaluate dominant TFs regulating the state-specific chromatin accessibility programs in an unbiased manner, we used TFModisco to analyze the ChromBPNet contribution scores within the top 10,000 ATAC-seq peaks of each state-specific accessibility topic delineated by MIRA (Figure S2B and S4A). TFModisco is a motif discovery algorithm that first identifies seqlets and performs iterative meta-clustering to aggregate seqlets with similar contribution score weight matrices to generate *de novo* motifs^49^. In the final step, the best match for each *de novo* motif pattern is determined by comparing it to known Jaspar TF motifs. We additionally merged related motif hits in each motif family (see Table S7) and then quantified the seqlet frequency across the various chromatin accessibility topics (Figure S4B, see representative TF family *de novo* motifs in Figure S4C). This enabled unbiased and comprehensive delineation of TF families acting on cCREs that were reflective of specific accessibility topics and B cell states (Figures 4C and S4B). Since many different TFs within a family can recognize the same motif, we also analyzed the transcript levels of all cognate TFs within each motif family (Figure S4D, Table S8). The combined analysis provided fine-resolution predictions for which TFs are most likely responsible for driving the dynamic chromatin programs during human B cell activation and differentiation. The B cell state-specific ChromBPNet models provide a valuable catalogue of TF activity inferences across all candidate *cis*-regulatory elements (cCREs) observed in diverse B cell states including naïve, ActB, GCBC and PB (https://tinyurl.com/BcellMultiomeTracks). These orthogonally derived state-specific TF activity predictions closely aligned with corresponding TF GRN centrality scores (Figure S2D). Strikingly, the TFModisco analysis, which was solely based on state-specific contribution score tracks, identified *de novo* motifs in the GC and PB topics that were matched with IRF4/IRF8 composite elements (Figure 4C, S4B). Specifically, the GC-1 topics contained AP-1::IRF (AICE) CWM motifs which are cooperatively bound by BATF and IRF4/IRF8 and the GC-2 topics contained ETS::IRF (EICE) CWM motifs which are cooperatively bound by PU.1/SPIB and IRF4/IRF8. In contrast, the PB topic was enriched for IRF::IRF (ISRE) CWM motifs which are bound by IRF4/IRF8 homo- or heterodimers^11,46^.

We next used a composite element motif scanning tool developed by us, CEseek^50^, to identify all instances of each of the forementioned CEs within their respective accessibility topics and compared the contribution score signals between cell states. As expected, the AICE-containing GC-1 topic (#1) peaks were more highly accessible in the GC-1 state compared to the PB state and EICE-containing GC-2 topic (#15) peaks were more accessible in the GC-2 state compared the PB state (Figure 4D). Conversely, the ISRE-containing PB topic (#7) peaks were more accessible in the PB state compared to the GC states. The aggregated contribution scores revealed substantial increases in the predicted contribution at AICE motifs in the GC-1 state and ISRE motifs in the PB state (Figure 4E). Interestingly, although the predicted contribution of EICE motifs was higher in the GC-2 state, they were also predicted to contribute in the PB state albeit with an altered base pair CS profile with less emphasis on the ETS half of the composite element. This was consistent with the increased activity of PRDM1 which is up-regulated in PBs and known to compete for binding of PU.1/SPIB at EICE motifs in murine B cells^46^. Thus, by analyzing B cell state-specific ChromBPNet models, in an unbiased as well as directed manner, we tested the hypothesis that the counterbalance between IRF4/IRF8 and their binding partners at AICE, EICE and ISRE motifs plays a critical role in determining human B cell fate choice. The results provide compelling orthogonal support for the hypothesis, as unlike MIRA-Cell Oracle GRN models, the ChromBPNet models infer TF centrality based on predicted single-nucleotide contributions to chromatin accessibility, independent of their relation to gene expression profiles.

### Single-cell perturbation analysis reveals combinatorial control of B cell fate choice

**Figure 5.**
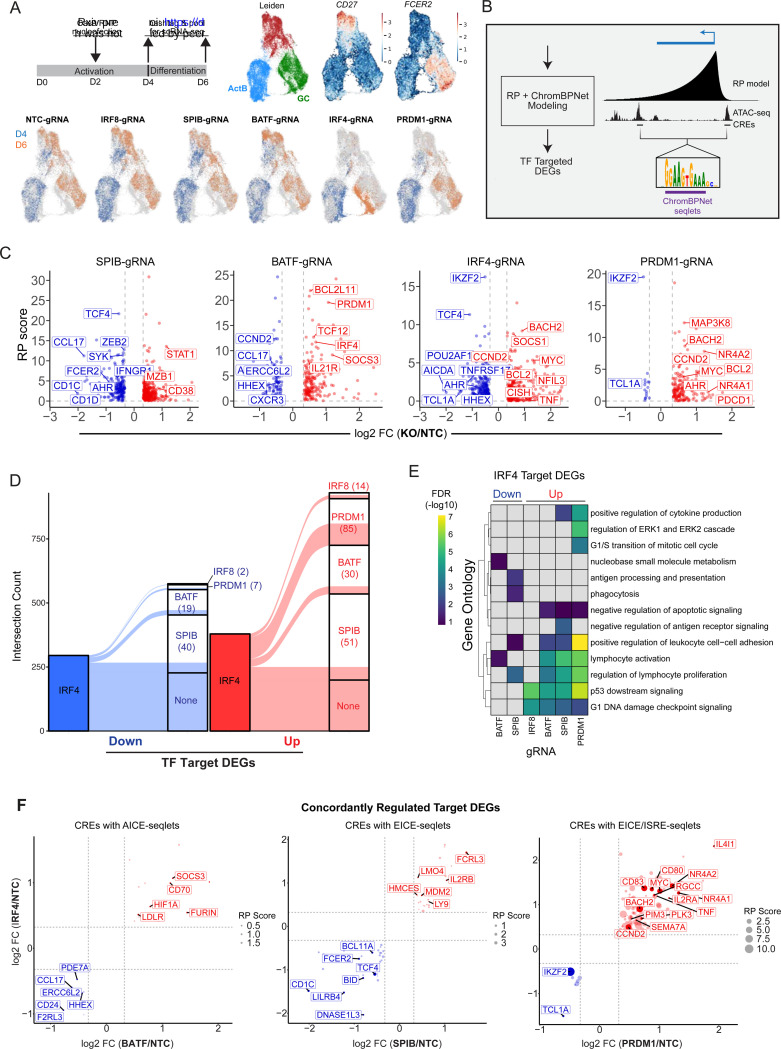
Single-cell perturbation analysis of counteracting PB and GCBC TFs. (A) Experimental strategy to profile TF CRISPR perturbations by scRNA-seq (*top left*). Naïve B cells (Donor 1) were activated for 2 days before nucleofecting with RNP complexes, re-culturing and harvesting cells at D4 and D6 for sample hashtagging and pooling for scRNA-seq. UMAP projections of aggregated cells (D4, D6) with their Leiden cluster labels and normalized expression of PB-marker gene *CD27* and GC-marker gene *FCER2* (CD23) (*top right*). UMAP projections displaying cells with the indicated gRNAs harvested at the indicated timepoints (D4, D6). (B) Computational strategy for identifying TF regulated target genes by connecting DEGs with ChromBPNet TF motif seqlets via gene-specific RP models. (C) Volcano plots displaying mean log2 fold change (FC) and RP scores (summed RP values of all ATAC-seq peaks containing ChromBPNet seqlets that correspond to binding sites of perturbed TF) for each DEG which is concordantly differentially expressed (|FC| > 1.25 and FDR < 0.05) between experimental replicates in the context of indicated TF KO. Dotted gray lines mark |FC| of 1.25. (D) Intersection of IRF4 KO DEGs with other TF KO DEGs (numbers indicate concordant co-regulated DEGs). (E) Heatmap displaying gene ontology pathway analysis of IRF4 co-regulated DEG modules (FDR, false discovery rate). (F) Plots displaying IRF4 co-regulated genes with its partners BATF, SPIB and PRDM1. Only concordantly regulated DEGs which are linked to at least one shared composite element motif seqlet (AICE, EICE, ISRE) are included. Sizes of the circles indicate the RP score for the respective composite element seqlets that are associated with the indicated target gene. Dotted gray lines mark |FC| of 1.25.

The ChromBPNet analyses (Figure 4E), combined with the simulated and experimental TF perturbation screens (Figure 3E), strongly supported the hypothesis that the GC and PB cell fates are regulated by distinct cooperative and antagonistic regulatory activities of IRF4 and the partner/cooperating TFs, IRF8, BATF, SPIB and BLIMP1 via their binding to AICE, EICE and ISREs. To test this hypothesis at genomic resolution, we analyzed the consequences of knocking out *IRF4*, *IRF8*, *SPIB*, *BATF* or *PRDM1* (BLIMP1) in activated B cells by scRNA-seq. The experimental design involved initiating the Cas9-RNP editing at Day 2 and performing scRNA-seq at Day 4 and 6 as the cells underwent specification into PB or GC fates (Figure 5A, top left). Following MIRA gene expression topic modeling and Leiden clustering, we identified three major clusters corresponding to ActB, GCBCs and PBs (Figure 5A, top middle). The ActB cluster was exclusively derived from the Day 4 timepoint and expressed high levels of *BATF*, *SPIB*, and *AICDA* but low levels of the GCBC genes *FCER2* (CD23) and *LMO2* (Figure 5A top right, Figure S5A). In contrast, the GCBC and PB clusters were primarily composed of Day 6 cells and expressed high levels of their respective marker genes (*AICDA*, *FCER2*, *LMO2* for GCBCs and *CD27*, *JCHAIN* and *MZB1* for PBs). In agreement with the immunophenotyping analysis (Figure 3D), IRF8, BATF, and SPIB KOs displayed moderate skewing towards PB differentiation (Figure S5B). In contrast, PRDM1 and IRF4 KO cells were severely impaired for PB formation and biased towards the GC fate. Notably, whereas PRDM1 KO cells generated GCBCs that were indistinguishable from their control counterparts, IRF4 KO cells were nearly completely segregated from other cells in the GCBC cluster, likely representing a stalled GCBC transition state. This was consistent with the duality of IRF4 action in controlling both GCBC and PB fates^11,13^.

To uncover gene programs that are cooperatively or antagonistically regulated by these TFs, we analyzed the differentially expressed genes (DEGs) within the Day 4 ActB cluster. By focusing our analysis on bipotent ActB cells we increased the likelihood of detecting TF-controlled gene modules that initiate programming of GCBC or PB fates. Profiling of the perturbed transcriptomes 48 hours post-RNP nucleofection also minimized secondary effects, thereby enriching for direct TF target genes. We restricted our analysis to DEGs that were concordantly significantly up- or down-regulated (|FC| > 1.25, FDR <= 0.05) between two replicates (Pearson’s correlation r = 0.92 to 0.97) (Figure S5C). In keeping with the distinctive stalled GCBC transition state manifested by IRF4-KO cells, they displayed the most statistically significant DEGs (n=720) followed by SPIB-KO (n=594), BATF-KO (n=297), PRDM1-KO (n=217) and IRF8-KO (n=33).

To identify genes that were likely to be directly regulated by each of the TFs within their cognate sets of DEGs, we integrated the MIRA RP models with those from ChromBPNet. The former predicted cCREs for each DEG based on the single-cell correlations between gene expression levels and accessibility of the cCREs (Figure 2A) whereas the latter predicted sites of TF binding and action within those cCREs based on base-pair contributions to chromatin accessibility profiles (Figure 4A). To infer which cCREs are regulated by IRF4 or one of its partner TFs in activated B cells, we used the Day 3 ActB ChromBPNet model to identify cCREs that contain seqlets whose contribution weight matrices were matched with relevant annotated TF motifs (see Methods). Unlike simple motif scanning, this approach discerned which TF motif instances likely contribute to the accessibility of a given cCRE in a particular cell state. We identified all seqlet instances within the union set of ATAC-seq peaks for AP-1 (BATF), ETS (SPIB), ISRE (IRF4/IRF8), PRDM1 (BLIMP1), AICE (BATF::IRF4), EICE (SPIB::IRF4/IRF8, BLIMP1) and PRDM1::IRF (BLIMP1/IRF4/IRF8) motifs (Figure S5D). These motif seqlets were then intersected with cCREs linked to DEGs via the RP models. This enabled quantitation of the number of seqlet-cCREs and their regulatory potential, resulting in a RP score for each TF-gene pair (sum of individual seqlet-containing cCRE RP values within a given RP model) (Figure 5C). For the delineation of direct target genes of a given TF, we identified DEGs of that TF, each of which contained at least one cognate ChromBPNet seqlet within a RP model-linked cCRE. This resulted in delineation of IRF8 (n=27), SPIB (n=562), BATF (n=289), IRF4 (n=629), and PRDM1 (n=201) regulated genes that were direct targets (Table S9). To evaluate the quality of the TF-target gene linkages in the MIRA assembled base-GRN model (Figure 2), we tested for enrichment of TF KO DEGs in corresponding TF-target gene sets (Figure S5E). This revealed that IRF4- and BATF-KO DEGs were enriched in the MIRA-predicted base-GRN TF-gene linkages (IRF4, odds ratio = 1.4 and p = 9.9e-4; BATF, odds ratio = 1.3 and p = 6.2e-2). Additionally, for all four analyzed TF-gene linkages, the MIRA assembled base-GRN model, derived from ChIP-seq peak ISD modeling, performed substantially better than the default Cicero assembled base-GRN model, derived from TF motif enrichment co-accessibility modeling.

Based on our hypothesis that IRF4 co-regulates divergent B cell programs through its combinatorial activity with partner TFs, we intersected IRF4 target genes with those of its putative binding partners. We identified statistically significant overlaps (FDR < 0.001) between the up or down IRF4 targeted genes and those of its partners (38% and 48% of PRDM1-down and -up DEGs; 18% and 16% of BATF-down and -up DEGs; 19% and 17% of SPIB-down and -up DEGs) (Figure 5D, Table S10). Gene ontology analysis revealed enrichment in immune-related pathways for all IRF4 co-regulated genes except for IRF4/IRF8-downDEGs and IRF4/PRDM1-downDEGs which had very small sample sizes (Figure 5E, Table S11). IRF4 and BATF co-activated genes were enriched for pathways associated with DNA processing/repair (*AICDA*, *MACROD2*, and *PDE7A*) whereas IRF4 and SPIB positively regulated target genes were enriched for pathways associated with lymphocyte proliferation (*AHR*, *BID*, *SYK*, *LILRB4*, *CD24*) and antigen processing and presentation (*CD1C*, *FCER2*, *MPEG1*). In contrast, all IRF4 co-repressed gene sets were enriched in pathways associated with DNA damage responses and p53 signaling, suggesting IRF4 broadly cooperates with multiple partners to dampen the response to DNA damage that is induced in the GC state because of AID-induced somatic hypermutation and class-switch recombination. In contrast, IRF4 and PRDM1 (BLIMP1) co-repressed genes were uniquely enriched for G1/S transition (*MYC, CCND2, PLK3, MDM2, RGCC*) and regulation of lymphocyte proliferation, cell-cell interaction (*CD80, CD70, BACH2, IL2RA, SLAMF1, CD274, IL4I1, TNF, SEMA7A*).

Next, we quantified the number of cCREs linked to each co-regulated target gene set that contained ChromBPNet seqlet predictions for shared composite elements for the respective TF pairs (Figure 5F). Strikingly, the vast majority of IRF4 and PRDM1 (BLIMP1) co-repressed genes had higher RP scores. This strongly suggested that IRF4 and BLIMP1 bind to ISRE and EICE motifs within multiple CREs associated with genes that encode regulators of B cell proliferation and GCBC formation culminating in their coordinated repression.

### Cell cycle-coupled transcriptional network drives B cell fate choice

**Figure 6.**
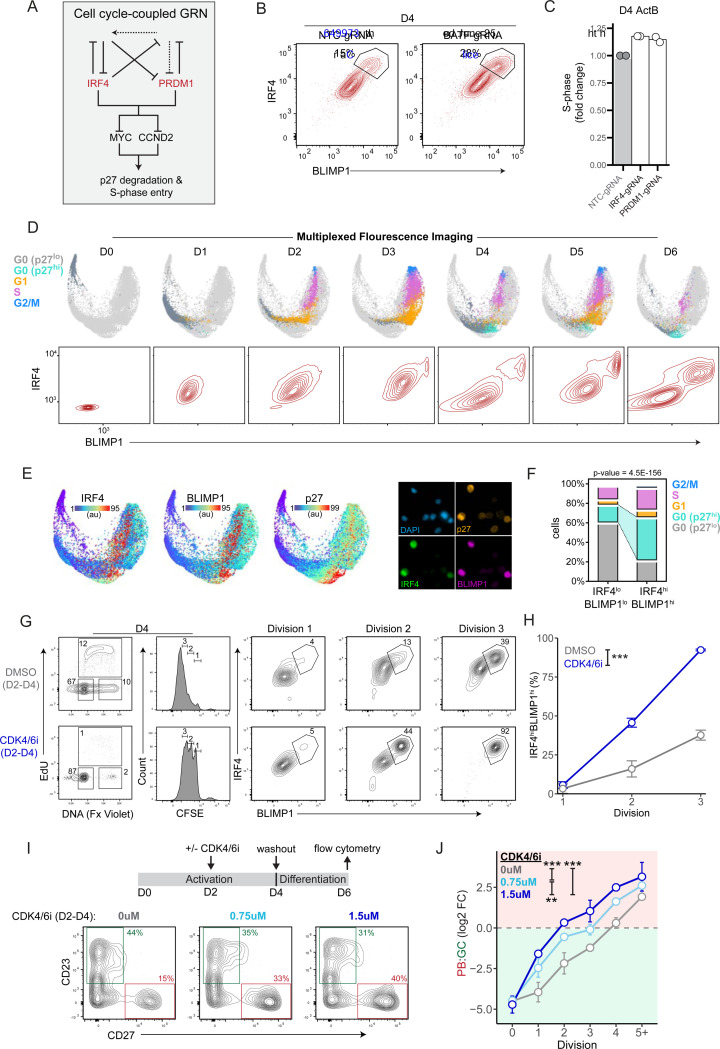
Cell cycle-coupled transcriptional network drives B cell fate choice. (A) Sub-GRN displaying regulatory interactions between GC and PB fate determining TFs and key cell cycle target genes. Solid lines indicate experimentally validated transcriptional repression edges (Figure 5C). Dotted lines represent edges based on prior knowledge. (B) Flow cytometry analysis of impact of BATF KO on expression of IRF4 and BLIMP1 (Day 4). Plots display indicated protein distribution in cells receiving NTC- or BATF-gRNAs. (C) Impact of IRF4 or PRDM1 KO on B cell S-phase frequency determined by applying SCANPY ‘score_genes_cell_cycle’ to the TF perturbation scRNA-seq data (Figure 5A). (D) Naïve B cells were differentiated *in vitro* as described in Figure 1A and harvested every 24h for fixation and multiplexed fluorescence imaging to simultaneously measure expression of cell cycle regulators (see Methods), IRF4 and BLIMP1. Integrated cell cycle manifold projections are displayed with cells from each timepoint annotated by cell cycle state (*top*) with distribution of IRF4 and BLIMP1 levels for each timepoint (*bottom*). (E) Cell cycle manifold projections with IRF4, BLIMP1, or p27 levels are displayed (*left*) along with representative fluorescence images for IRF4, BLIMP1, and p27 of the same field of cells (20X). (F) Comparison of cell cycle phase distributions within IRF4^lo^BLIMP^lo^ and IRF4^hi^BLIMP^hi^ cells at D4. Fisher’s exact test was used to test for significance in alteration of G0 (p27^hi^) frequency (odds-ratio^hi/hi vs. lo/lo^ = 3.6). (G) Effect of slowing the cell cycle on IRF4 and BLIMP1 levels. Flow cytometry analysis of CFSE-labeled activated B cells treated with DMSO or the CDK4/6 inhibitor (CDKi), PD0332991 (2uM), between D2-D4. (H) Quantification of IRF4^hi^BLIMP^hi^ cells as a function of cell division at D4 (*n* = 3 experimental replicates; ***P < 0.001 by two-way ANOVA). (I) Effects of slowing the cell cycle on final PB-GC frequencies. Flow cytometry analysis (D6) of PB (CD27^hi^CD23^lo^) and GC (CD27^lo^CD23^hi^) cells generated after culture of activated B cells with DMSO or CDKi (D2-D4) and re-culturing in absence of the inhibitor for two days in differentiation media (see Figure 1A). (J) Quantification of PB/GC log2 fold change (FC) as a function of CDKi concentration and cell division number. Flow cytometry data acquired at D6 was used for analysis (*n* = 3 experimental replicates; **P < 0.01 and ***P < 0.001 by two-way ANOVA with Tukey’s multiple comparison test).

Strikingly, our single-cell perturbation profiling of IRF4 and its partner TFs uncovered reciprocal negative feedback loops involving the pro-GC TF, BATF, and the pro-PB TFs, IRF4 and PRDM1 (Figure 6A). Notably, IRF4 and PRDM1 counter-repressed *BATF* and its upstream activator *BACH2*, respectively. This regulatory module predicted that BATF depletion would elevate the levels of IRF4 and BLIMP1. In keeping with this prediction, BATF KO cells displayed increased IRF4 and BLIMP1 protein expression at Day 4 (Figure 6B). Intriguingly, the perturbation analysis also revealed that IRF4 and BLIMP1 co-repress *MYC* and *CCND2* (cyclin D2) which promote G0/G1-S phase transition in part via p27 degradation^51,52^. Thus, perturbation of IRF4 or PRDM1 would be expected to result in increased frequency of cells in S-phase. Analysis of the single-cell perturbation experiments (Figure 5A) revealed that depletion of either IRF4 or PRDM1 led to increases in the frequencies of cells with an S-phase signature on Day 4 (Figure 6C). Together, these results suggested that the balance between counter-acting pro-GC TFs (BATF/BACH2) and pro-PB TFs (IRF4/PRDM1) also controls the cell cycle dynamics of multipotent ActB cells.

Based on these considerations, we used a multiplexed single-cell imaging approach to temporally profile 21 different cycle regulators concurrently with IRF4 and BLIMP1 protein levels in CFSE-labeled cells (D0-D6). We used manifold learning to project cells in low-dimensional space according to their levels of key cell cycle regulators (Figure 6D, upper panels). Importantly, this approach enabled high-resolution visualization of continuous cell cycle states at the single-cell level which are highly correlated with those observed by live-cell imaging^53^. In agreement with the EdU labeling experiments (Figure S1A), a small fraction of activated B cells entered S-phase at D1 before undergoing mitosis at D2 (Figure 6D). Concurrently, IRF4 levels increased rapidly at D1, (Figure 6D, lower panels). By D3, nearly all cells were cycling in G1/S or G2/M and expressed intermediate levels of IRF4 and BLIMP1 (Figures 6D and S6A). Strikingly, at D4, as IRF4^hi^/BLIMP1^hi^ cells first emerged, a large fraction of cells had exited into a G0 state. Coincident up-regulation of IRF4 and BLIMP1 expression was associated with the highest p27 levels in a distinguishable cluster of G0 cells (Figure 6E). To quantify this relationship, we compared the distribution of cell cycle states of IRF4^lo^/BLIMP1^lo^ and IRF4^hi^/BLIMP1^hi^ cells at D4. This revealed that IRF4^hi^/BLIMP1^hi^ cells were 3.6 times more likely to exist in a p27^hi^-G0 state compared with IRF4^lo^/BLIMP1^lo^ cells (Fisher’s exact test, p-value = 4.5E-156) (Figure 6F). Given that IRF4 and BLIMP1 combinatorially repress the p27 repressors (*MYC* and *CCND2*) (Figures 5F and 6A), these results suggested that IRF4 and BLIMP1 up-regulation potentiates cell cycle exit into a G0 state by inducing high levels of p27 in activated B cells. Importantly, this quiescent G0 state is transient as most cells (~82%) re-enter the cell cycle at D5 upon restimulation with CD40L and cytokines (Figure S6A).

The results above raised the possibility that the duration of the G0/G1-S phase transition could feedback into the GRN module by controlling the accumulation IRF4 and BLIMP1, thereby increasing the probability of PB specification. Such an auto-regulatory positive feedback loop - involving graded expression of a cell fate determining TF and cell cycle lengthening - has been proposed to modulate specification of alternative hematopoietic cell fates^54,55^. To test if extending the G0/G1 phase increases IRF4 and BLIMP1 accumulation, we cultured activated B cells in the presence of the CDK4/6 inhibitor (PD0332991, 2 μM). CDK4 and CDK6 are targets of p27 and thus inhibiting them mimics the increased p27 levels observed in IRF4^hi^/BLIMP1^hi^ cells. The inhibitor was added after B cell activation, between D2-D4, before quantifying IRF4 and BLIMP1 levels by flow cytometry. In the absence of the inhibitor, D4 activated B cells retained moderate proliferation rates (12% EdU+) and most cells had completed three to four cell divisions (Figure 6G). CDK4/6 inhibition (CDK4/6i) dramatically decreased the frequency of proliferating cells at D4 (1% EdU+) and resulted in a pronounced shift in the cell division distribution with majority of cells completing 1–3 cell divisions. As expected, IRF4 and BLIMP1 levels increased as a function of cell division number. Importantly, CDK4/6 inhibition significantly increased the frequency of IRF4^hi^/BLIMP1^hi^ at cell division 2 (46% vs. 16%) and division 3 (92% vs. 38%) (Figure 6H). Thus, G0/G1 phase lengthening in activated B cells enhanced the coordinated up-regulation of IRF4 and BLIMP1, resulting in a self-reinforcing feedback loop.

Given that B cell fate divergence begins between D2-D4 and that IRF4 and BLIMP1 drive PB specification (Figures 1A, 1C and 2A), we hypothesized that the frequency and duration of a transient G0/G1 phase during this period determine the final proportions of PBs and GCBCs. To test this possibility, we transiently promoted G0 phase lengthening by culturing cells in sub-optimal concentrations of CDK4/6i (0.75 μM or 1.5 μM) between D2-D4 before washing out the inhibitor and re-culturing for two days (Figure 6I). Flow cytometry analysis of CD27 (PB-marker) and CD23 (GC-marker) levels revealed that G0/G1 phase lengthening resulted in a dose-dependent increase in the final PB:GC ratio. Additionally, this dose-dependent PB-skewing by G0/G1 phase lengthening was also observed on a per cell division basis (Figure 6J). These results demonstrated that cell cycle speed rather than cell division number *per se*, modulates B cell fate probabilities. Specifically, G0/G1 phase lengthening promotes PB fate specification.

These findings generated an apparent paradox. If slower cell cycle speed favors PB commitment, why does PB differentiation probability increase as a function of cell division number^19,20^ and why do PBs ultimately complete more cell divisions than GCBCs (Figure 1B). We hypothesized that during the activation and clonal expansion phase, the likelihood of entering a transient G0 state increases as function of cell division, thereby indirectly linking division history to B cell fate choice. To investigate this, we measured p27 levels as a function of cell division in D3 activated B cells. This revealed that like IRF4 and BLIMP1, p27 but not RB increased as a function of cell division (Figure S6B). Furthermore, cells that completed the most divisions (CFSE-low) by D3-D4 were less likely to be in S-phase (EdU+) when compared to CFSE-high cells at the same timepoints (31% vs. 71% at D3 and 2% vs. 20% at D4) (Figure S6C). Together, these results suggested that activated B cells which more rapidly enter the first cell cycle (D2) and thus complete more cell divisions by D4 are more likely to up-regulate p27 and experience a prolonged G0/G1 phase which favors PB commitment. If this were the case, then one would expect the difference between the frequencies of PB and GCBC fated cells to be determined during the clonal burst that occurs during the first four days. To test this, we labeled cells at D0 or D4 with the proliferation dyes -CellTrace Violet (CTV) at D0 and CellTrace Yellow (CTY) at D4- to analyze the ‘post-D0’ and ‘post-D4’ cell division numbers for GCBCs and PBs at D5 and D6 (Figure S6D). Strikingly, PBs emerging at D5 were highly enriched among cells that completed the most ‘post-D0’ cell divisions, typically four to six. However, these PBs completed the same number of ‘post-D4’ cell divisions as GCBCs, suggesting that PBs originate from cells which complete more cell divisions between D0-D4. Collectively, these results suggested that activated B cells which enter the cell cycle more rapidly are more likely to enter a prolonged G0/G1 phase after clonal bursting, which enhances IRF4 and BLIMP1 expression and biases their fate commitment toward PB over GCBC.

## DISCUSSION

In this study, we have assembled, simulated, and tested human B cell GRN models that span temporally resolved activation states and bifurcated PB and GCBC fates. In so doing, we revealed the dominant and reciprocal dynamics of IRF4 in regulating the B cell fate bifurcation via its interaction partners at IRF composite elements and ISREs in GCBC and PB states, respectively. Furthermore, we uncovered a cell cycle-coupled transcriptional regulatory module that controls the balance between PB and GCBC differentiation. We propose that such self-reinforcing regulatory loops between cell cycle and TF determinants enable signaling-dependent tuning of probabilistic fate decisions within multipotent progenitor compartments by growth factor or cytokine signaling. The integrated experimental and computational analyses provide an extensive catalogue of quantitatively prioritized B cell state-specific TF-CRE and TF-gene linkages. The base-pair resolution ChromBPNet models will be particularly useful for understanding the molecular underpinnings of state-specific gene regulation and can be applied to predict the effects of non-coding genetic variants associated with B cell-mediated autoimmune diseases^48,56^.

The expansion of single-cell analyses that simultaneously profile transcriptional and chromatin landscapes has propelled the widespread application of GRN modeling across diverse developmental and cellular contexts^6^. However, GRN modeling has been limited by the following issues that have been addressed herein. First, instead of relying solely on co-accessibility patterns to connect cCREs and target gene promoters, we apply MIRA RP modeling which infers the regulatory potential of cCREs neighboring a gene based on co-variation of cCRE accessibility and gene expression patterns. Second, rather than predicting TF binding and action on cCREs simply based on TF motif enrichment and/or occurrence, which cannot distinguish different structurally related TFs, we utilize the Cistrome DB database as a source of thousands of uniformly processed ChIP-seq datasets. MIRA ISD modeling is then used to identify likely TF-gene connections based on simulating the removal of cCREs with evidence of TF binding in related cellular contexts. Third, rather than assigning the same TF-gene linkages to all cell states of a given system, we applied CellOracle GRN modeling to transform cell-state agnostic and qualitative linkages into state-specific and quantitative TF-gene linkages. Collectively, integration of these three modeling approaches allows us to simultaneously anchor the TF-gene linkages to physical TF-specific binding events and to infer the strength and directionality of linkage reconfigurations during a dynamic differentiation process. Importantly, the improved performance of GRN models based on an integration of MIRA with CellOracle was demonstrated with two types of experiments that tested the impact of TF perturbations on cell fate determination as well as on their target genes (see below).

The assembled GRNs provide comprehensive predictions of TFs and their target genes that drive human B cell responses and account for their functions. Previously, such predictive modeling has not been systematically tested with corresponding experimental perturbations. Here, we tested the impact of perturbing 32 TFs that were predicted to differentially impact the generation of GCBCs and PBs. These results represent the first comprehensive and quantitative testing of a large set of TFs in controlling the generation of human GC and PB cells. The integrated computational framework (MIRA and CellOracle) demonstrated excellent concordance between in-silico-perturbations and their experiment analyses demonstrating the quality of the assembled GRNs. Notably, CellOracle and other *in silico* perturbation approaches have performed less well in benchmarking analyses^57^. This has primarily been attributed to limitations of modeling assumptions for in-silico perturbations. However, our analyses suggested that higher-quality GRN assemblies can significantly mitigate issues with corresponding modeling assumptions. In addition to demonstrating the effectiveness of our GRN model predictions, the results from the predict-perturb-test framework^58^ both corroborate and substantially extend our previous knowledge of murine B cell GRNs^59–61^. Our simulations coupled with experiments provide an even more compelling demonstration that IRF4 and PRDM1 are the dominant regulators of PB differentiation. In contrast, the GCBC fate is controlled by many different TFs (BATF, PAX5, MEF2C, SPIB, NFKB1). A notable exception was BCL6 that was predicted to have strong effects on the GC:PB ratio, but our experiments detected only mild phenotypic consequences. This was likely due to continuous CD40 stimulation in our culture system which has been shown to limit sustained high expression of Bcl6 in GC precursors^62^. The absence of single TF perturbations in our experimental system that dramatically decrease the human GC:PB ratio mirrors results of CRISPR screens in murine B cells^63–65^, suggesting GCBC formation is likely regulated by multiple, partially redundant factors.

The specification of divergent PB and GC fates has been suggested to be initiated by cross-antagonism between IRF4 and the related TF, IRF8, and their interactions with various partner TFs at composite elements^13,44,45^. Our unbiased high resolution genomic analysis, using orthogonal computational tools (MIRA+CellOracle and ChromBPNet), of multiome datasets of human B cell fate dynamics provided strong evidence for this regulatory model. Here, by systematic profiling of perturbations of IRF4/IRF8 and their interacting partners, we delineated gene programs that are co-regulated by various combinatorial pairs in activated B cells that could give rise to PBs or GCBCs. The most striking co-regulatory relationship was between IRF4 and PRDM1, which we found initially function to co-repress genes associated with B cell activation, survival and proliferation programs. This contrasts with the cell migration and antibody secretion gene programs that IRF4 and PRDM1 regulate during PB differentiation^13,46^. The results strongly suggest that the specification of the PB fate is initiated by repression of gene programs that are needed to sustain the GC fate.

How might IRF4 and BLIMP1 cooperate to repress genes in activated B cells? IRF4 and BLIMP1 bind very similar motifs (5’-GAAA-NN-GAAA-3’ vs. GAAA-GT-GAAAGT-3’) and exhibit substantial overlap in binding sites by ChIP-seq^46^. Previous work has demonstrated that BLIMP1 can compete with IRF4 and PU.1 for binding at EICE motifs (5’-GGAA-NN-GAAA-3’). However, it remains possible IRF4 and BLIMP1 can bind cooperatively at ISRE motifs (5’-GAAA-NN-GAAA-3’)^13^. Compared to binding the EICE motif as a heterodimer with PU.1, IRF4 displays much lower binding affinity for ISRE motifs in vitro and requires high concentrations for optimal binding^13^. IRF4 binding to ISREs may be enhanced by cooperativity with BLIMP1 and explain why both TFs are required in the ActB cells to co-repress genes linked to ISRE-related motifs.

How is the up regulation of IRF4 and BLIMP1 coordinated during PB fate specification? Notably, while IRF4 and PRDM1 appear to reinforce each other’s expression in the PB state,^46^ we did not find any experimental evidence for such reciprocal positive feedback in the ActB state. Instead, the GRN model and its testing revealed that *IRF4* and *PRDM1* are repressed by a common up-stream regulator, BATF that promotes the GCBC fate. Thus, as BATF expression decreases over time, IRF4 and PRDM1 are simultaneously up regulated which we found can additionally trigger two positive feedback loops further elevating their expression. One such feedback loop involves repression of the negative regulators BATF and BACH2. The second feedback loop involves G0/G1 elongation by cooperative repression of the *MYC* and *CCND2* genes that in turn augments accumulation of the two TFs. The molecular mechanism by which a prolonged G0/G1 phase results in the increased expression of IRF4 and PRDM1 remains to be elucidated but may involve diminished rates of degradation of the two proteins which are highly stable under certain contexts^26,66^.

The cell-cycle coupled BATF/IRF4/BLIMP1 GRN module we describe herein provides new insights into the long-standing question about the relationship between cell division and B cell fate^19,20^. We found that IRF4 and BLIMP1 co-repressed genes were enriched for the G0/G1->S transition and proliferation. Furthermore, depletion of either TF increased the frequency of S-phase cells. We therefore propose that coordinated upregulation of IRF4 and PRDM1 above a threshold triggers transient G0 pausing prior to PB fate specification. In turn, extending G0/G1 enhances the up regulation of IRF4 and BLIMP1 and leads to increased frequency of PB generation at the expense of GCBCs. Thus, we propose a model in which cell cycle rates, not cell division number *per se*, determine the probabilities of PB vs. GCBC fate determination. This regulatory model is consistent with a previous report in murine B cells in which extending G0/G1 by CDK inhibition increased PB differentiation *in vitro*^24,67^. Conversely, depletion of the CDK inhibitor p18 reduced PC formation and enhanced GC formation *in vivo*^68^. Cell cycle slowing has previously been proposed to explain why diminishing CD40 stimulation increase PB differentiation rates^18^. This may explain our observations that the generation of the GC fate is highly dependent on CD40L concentrations between Days 4 and 6, as it likely maintains shorter G0/G1->S transitions which favor GCBC formation. Our results substantially extend previous findings by uncovering new GRN-based mechanistic underpinnings and strongly suggest that molecular cooperativity between IRF4 and BLIMP1 promotes the initiation of an extended G0/G1 phase. Furthermore, we find that cell cycle lengthening triggers a positive feedback loop that accelerates the switching of cells to an IRF4^hi^BLIMP1^hi^ regulatory state and enhances the probability of PB specification, thereby uncovering a self-reinforcing regulatory module that couples cell cycle dynamics to B cell fate choice.

Signaling modulated pausing in G0/G1 phase has been observed across many developmental systems and may be a general mechanism for triggering differentiation events^69,70^. The IRF4/BLIMP1 coupled cell-cycle feedback loop we describe herein is reminiscent of a PU.1 coupled cell-cycle feedback loop which regulates the myeloid vs. B cell fate decision among hematopoietic progenitors^54,71^. As is the case for IRF4 and BLIMP1 in ActB cells, PU.1 levels control and are controlled by G0/G1 phase lengthening. Differences in TF protein stability could enable G0/G1 phase extensions to selectively elevate the levels of one fate regulator more dramatically than another. It has recently been demonstrated that down-regulation of MYC is required for normal PB differentiation, highlighting the importance of timing of gene silencing in B cells^72^. Our results suggest that timed decrease in MYC production rate is determined by the levels of IRF4 and BLIMP1 which we demonstrate cooperate to repress MYC transcription. One consequence of diminished MYC expression is the accumulation of p27, which has previously been proposed as a molecular timekeeping mechanism in oligodendrocyte differentiation^73^. Consistent with this possibility, we observed increases in p27 accumulation that were correlated with IRF4/BLIMP1 up-regulation and cell cycle exit. Importantly, consistent with our observations, that after their cell fate specification, PBs undergo several cell divisions, it has been shown that after PB commitment, PIM2-mediated p27 degradation enables cell cycle progression^74^.

Our results suggest that human B cells which enter the cell cycle more rapidly are more likely to experience an extended G0/G1 phase after a series of cell divisions. In this paused transitional state, both IRF4 and BLIMP1 accumulate above threshold levels and trigger PB commitment through two self-reinforcing feedback loops. One consequence of this model is that clones which have divided less are less likely to experience a prolonged G0/G1 phase and thus more likely to adopt the GC fate. Therefore, the pool of GC precursors will be predicted to be more diverse from the standpoint of their B cell repertoire. Coupling activated B cell division destiny to cell fate may serve to ensure that GC reactions are initiated by a more diverse pool of BCR clonotypes which can then undergo competition and affinity maturation. Additionally, the BATF/IRF4/BLIMP1 cell-cycle coupled feedback loop may also contribute to the association between BCR affinity and B cell fate. B cells with higher BCR affinities achieve greater cell division destinies upon stimulation and have increased PB:GC ratios^75^, raising the possibility that BCR signaling strength skews B cells fates partly by modulating cell division destiny and transient G0/G1 pausing. Intriguingly, T cell fates are also correlated with cell division speed, with slower cell divisions and elongated G1 phases associated with the formation of central memory precursors^76,77^. Given that BATF, IRF4 and BLIMP1 are also important regulators of T cell differentiation, it raises the possibility that the cell cycle coupled regulatory module described in B cells may also operate to control T cell responses.

## RESOURCE AVAILABILITY

### Lead Contact

Further requests and information concerning this study should be addressed to the lead contact, Harinder Singh (harinder@pitt.edu).

### Materials Availability

This study did not generate new unique reagents.

### Data and Code Availability

All sequencing data has been deposited to the IGVF Data Portal (https://data.igvf.org/): IGVFDS4024LHFB, IGVFDS7859EYQR, IGVFDS8967LLYA, IGVFDS8822AFTR, IGVFDS9764OTVQ, IGVFDS8419ZMNL, IGVFDS2610IANK, IGVFDS7476HGYD, IGVFDS5867EPVA. Supplemental tables, flow cytometry and single-cell multiplexed imaging datasets have been deposited at Zenodo: 10.5281/zenodo.15213342. Scripts and associated documentation necessary to reproduce the analysis of genomics datasets have been deposited to GitHub: https://github.com/pitt-csi/humanBcell_GRN.

## METHODS

### Primary B cell purification and in vitro differentiation

Peripheral blood mononuclear cells (PBMCs) from healthy donor leukapheresis products (StemCell Technologies) were isolated by Ficoll density centrifugation. Donors were recruited with institutional review board approval and informed consent by STEMCELL Technologies. Naïve B cells were purified by negative selection using magnetic bead separation (EasySep Human Naïve B Cell Isolation or Memory B Cell Isolation Kits, STEMCELL Technologies). Purified Naïve B cells were labeled with either 2μM CFSE for 10 minutes at 37°C or 5μM CellTrace Violet (CTV) for 20 minutes at room temperature before washing with complete RPMI. Naïve B cells were centrifuged and resuspended at 7.5 × 10^5^ cells/mL in the activation media [RPMI-GlutaMAX with 10% heat-inactived FBS, 1X Penicllin-Streptomycin, 2.6ug/mL of anti-IgM (Jackson ImmunoResearch), 400ng/mL CD40L (Biolegend), 2ng/mL IL-2 (Biolegend), 1ug/mL CpG ODN-2006 (Miltenyi)]. Naïve B cells were cultured in 24-well plates for four days before harvesting and washing with complete RPMI. After washing and centrifugation, activated cells were re-suspended at 4.5 × 10^5^ cells/mL in differentiation media [RPMI-GlutaMAX with 10% heat-inactived FBS, 1X PenStrep, 400ng/mL CD40L (Biolegend), 2ng/mL IL-2 (Biolegend), 5ng/mL IL-4 (Biolegend), 12.5ng/mL IL-10 (Biolegend). All centrifugation steps were performed at 300xg for 10 minutes at room temperature.

### Flow cytometry procedures and analysis

Cells were incubated with human Fc block (Biolegend) for 5 minutes at room temperature before staining with cell surface antibodies in FACS buffer [PBS with 2% FBS and 1mM EDTA] with 1X BD Brilliant Stain buffer (BD Biosciences) for 30 minutes on ice. Cells were washed with FACS buffer twice before resuspending in 1X fixation buffer (eBioscience Foxp3 / Transcription Factor Staining Buffer Set) and incubating for 20 minutes at room temperature. Cells were centrifuged at 600xg for 5 minutes before washing with 1X permeabilization buffer (eBioscience Foxp3 / Transcription Factor Staining Buffer Set), centrifuged again and resuspended in 1X permeabilization buffer with 2% rat serum blocking solution. Cells were incubated for 15 minutes at room temperature before adding intracellular antibodies and incubated overnight at 4C. Cells were then washed three times with 1X permeabilization buffer before resuspending in FACS buffer and analyzing on an Attune NxT (Thermo Fisher Scientific) or an Aurora (Cytek). For EdU labeling experiments, cells were incubated with 10uM EdU (Thermo Fisher Scientific) for 1.5 hours prior to harvesting cells for fixation, cell surface staining, and permeabilization. EdU-labeled cells were then incubated with Click-iT reaction cocktail for 30 minutes at room temperature before washing in permeabilization buffer and resuspending in 1:1000 FxCycle Violet (Thermo Fisher Scientific). After a 30 minute incubation on ice, cells were analyzed by flow cytometry. See Table S12 for antibody details.

### Single-cell RNA-sequencing and BCR-sequencing procedure

Naïve B cells from Donor 1 were isolated and activated as described above every two days before harvesting cells asynchronous cultures on Day 6. Cells were washed with FACS buffer before removing dead cells by magnetic cell isolation (EasySep Dead Cell Removal Kit, STEMCELL Technologies). Live cells were incubated in Human TruStain FcX Fc Receptor Blocking Solution (Biolegend) for 10 minutes on ice before washing and resuspending in PBS with 10% FBS. Cells were then stained with TotalSeq-C anti-human hashtag oligonucleotide conjugated antibodies (Biolegend, see Table S12 for antibody details) for 30 minutes on ice. Cells were then washed three times with 10% FBS PBS before processing with Single-cell 5’ Kit v2 (10x Genomics) and Human BCR Amplification Kit (10x Genomics) according to manufacturer instructions. Resulting libraries were sequenced with a NovaSeq 6000 S1–100 cycle flow cell (v1.5) (Illumina).

### Single-cell RNA-sequencing and BCR-sequencing data analysis

FASTQ files were generated using CellRanger (v7.1.0) ‘mkfastq’ function and used as input into CellRanger (v7.1.0) ‘multi’ function using GRCh38 (2020) as reference genome. After demultiplexing using HashSolo, MIRA expression topic modeling was performed using the most highly variable genes. BCR-sequencing FASTQ files were processed with CellRanger (v7.1.0) ‘multi’ function using GRCh38 (2020) as reference genome (‘refdata-cellranger-vdj-GRCh38-alts-ensembl-7.0.0’). BCR clonotype analysis was restricted to clonotypes with at least two cells detected. Monte Carlo simulations (1,000) of expected number of clonotypes was performed by deriving a Poisson distribution of PB and GC clone sizes, randomly shuffling clonotype indices and randomly sampling the same number of cells that were sampled experimentally. P-values were calculated as previously described (You et al., 2023) by calculating a z-score comparing the observed and simulated counts for each clonotype category and calculating the probability of the observed counts being greater than or less than the expected (2 * (1 - cumulative distribution function of absolute z-score values)).

### Single-cell multiome procedure

For Donor 1 single-cell multi-omics, naïve B cells were isolated and activated as described above every 24 hours before harvesting asynchronous cultures on Day 6. Dead cells were removed by magnetic cell isolation (EasySep Dead Cell Removal (Annexin V) Kit, StemCell Technologies) and stained with fixable viability dye (FVD) (Thermo Fisher Scientific) for 30 minutes on ice before washing with FACS buffer and purifying by fluorescence-activated cell sorting (FACS). See Table S12 for antibody details. FACS-purified cells were washed with 1mL of PBS with 0.04% BSA twice before adding 100μL of chilled 10x Lysis Buffer [10mM Tris-HCl, 10mM NaCl, 3mM MgCl2, 0.1% Tween-20, 0.1% IGEPAL, 0.01% digitonin, 1% BSA, 1mM DTT, 1U/μL RNase inhibitor (Protector RNase inhibitor, Sigma)]. Cells were mixed by pipetting 10 times before incubating on ice for 3 minutes. 1mL of chilled 10x Wash Buffer [10mM Tris-HCl, 10mM NaCl, 3mM MgCl2, 0.1% Tween-20, 1% BSA, 1mM DTT, 1U/μL RNase inhibitor (Protector RNase inhibitor, Sigma)] was added to lysed cells and mixed five times by pipetting. Cells were washed three times with 10x Wash Buffer before resuspending in Diluted Nuclei Buffer [1X Nuclei Buffer (included in 10x Genomics Single-cell Multiome ATAC Kit A), 1mM DTT, 1U/μL RNase inhibitor] for counting and processing with Single-cell Multiome ATAC Kit (10x Genomics). Resulting libraries were sequenced on a NovaSeq SP 100 cycle flow cell (v1.5) (Illumina). For Donor 2 single-cell multi-omics, live cells from each timepoint were FACS-purified as described above and were frozen in Bambanker medium (FUJIFILM Irvine Scientific). Upon thawing, nuclei were isolated as described above and were processed according to an adapted version of the MULTI-seq protocol previously described^78^. Briefly, nuclei were resuspended in chilled PBS (750–1000 nuclei/μL) and aliquoted (180 μL per sample). A 1 μM Anchor-Barcode complex (final 50 nM) was added, mixed, and incubated on ice for 5 minutes, followed by the addition of 1 μM Co-Anchor (final 50 nM) with another 5 minutes incubation. Labeling reactions were quenched with 2% BSA in PBS and nuclei were centrifuged at 500xg for 4 minutes at 4°C. After resuspension, samples were pooled and centrifuged again before being resuspended in Diluted Nuclei Buffer for loading and processing according to Chromium Next GEM Single-cell Multiome ATAC + Gene Expression User Guide (10x Genomics) through the “Step 4: Pre-Amplification PCR step”. 10μL of the ‘Pre-Amplification SPRI Cleanup’ product was fractionated by incubating with 0.6X SPRI beads. The resulting supernatant containing the MULTI-seq barcodes was further purified by SPRI (3.2X) and isopropanol (1.8X). After performing two 80% ethanol washes and elution from SPRI beads, barcode DNA libraries were amplified using Kapa HiFi HotStart Ready Mix with universal I5 primer and TruSeq RPI primers. The resulting PCR product was purified by SPRI-clean up (1.6X), two 80% ethanol washes, and elution in EB Buffer. cDNA (RNA product) and gDNA (ATAC product) libraries were prepared according to Chromium Next GEM Single-cell Multiome ATAC + Gene Expression User Guide.

### Single-cell multiome data analysis

FASTQ files were generated using CellRanger (v7.1.0) ‘mkfastq’ function and used as input into CellRanger ARC pipeline (v2.0.2) using GRCh38 (2020) as reference genome for alignment, filtering, barcode counting, peak calling and molecule counting using the ‘count’ function. Outputs from each GEM well were aggregated using ‘aggr’ function. Using SCANPY^79^, cells were filtered with minimum of 200 genes/cell (with < 20% mitochondrial reads) and genes were filtered with minimum of 20 cells/gene. Variation due to cell cycle phase was regressed out using SCANPY^79^ ‘regress_out’ function using G1/S and G2/M gene lists previously described^80^. MIRA expression topic modeling was performed using the most highly variable genes (X genes). Following MIRA accessibility topic modeling, a joint embedding space was constructed by joining the transformed expression and accessibility topic spaces. HashSolo was used for demultiplexing Donor 2 sample timepoints based on MULTI barcode tag levels.

### STREAM psuedotime and differentiation trajectory inference

Pseudotime inference for B cell differentiation was performed using STREAM. Given the substantial differences in gene expression profiles between naïve B cells and other differentiation states, their inclusion introduced excessive noise in pseudotime inference. Therefore, naïve B cells were excluded from the analysis. Prior to running STREAM, gene expression counts were imputed using Palantir to enhance trajectory reconstruction, with diffusion map dimensionality set to 50 components for the male dataset and 10 components for the female dataset. Highly variable genes were selected by setting the loess fraction to 0.005 and retaining the top 80th percentile of genes for the male dataset, while for the female dataset, the loess fraction was set to 0.01 with the top 90th percentile of genes retained. Dimensionality reduction was performed using modified locally linear embedding (MLLE) for male samples and standard locally linear embedding (SE) for female samples.

Following dimensionality reduction, elastic principal graphs (EPGs) were fitted and optimized to construct branching trajectories. The parameters for fitting the graph were tuned to achieve biologically meaningful differentiation pathways. This approach enabled robust trajectory reconstruction, capturing key differentiation pathways while minimizing artifacts introduced by naïve cells. The resulting STREAM-derived pseudotime values were used to generate a differentiation gradient object using CellOracle’s ‘Gradient_calculator’ module. The resulting gradient object was then used to generate differentiation flow vector field graphs to model normal and perturbed differentiation trajectories.

### Gene Regulatory Network Inference with MIRA and CellOracle

MIRA regulatory potential (RP) modeling was performed for all highly variable genes with TSS information (4306 genes). The resulting gene-specific RP models were then used in conjunction with CistromeDB-processed ChIP-seq peaks^81^ (6912 datasets) to perform probabilistic *in silico* deletion (pISD) modeling. pISD scores for each TF-gene pair are calculated by removing all peaks that overlap given TF ChIP-seq peak set and measuring the change in the target gene’s RP model. For cases in which multiple ChIP-seq datasets are available for given TF, the top pISD score for each TF-gene pair was retained for downstream analysis.

To perform CellOracle GRN modeling, only TF-gene pairs with a pISD score among the top 5% of all scores were retained as binarized linkages which serve as ‘base-GRN’. This resulted in ~215 predicted target genes per TF. Only TFs present in the human TF database^82^ were included. For TFs which lack MIRA inferred linkages (due to ChIP-seq availability or quality), we incorporated co-accessibility-based TF-gene linkages inferred using Cicero (co-accessibility > 0.8) from the scATAC-seq data in the multiome. Cicero-derived linkages were added only for target genes present in the highly variable gene set from MIRA for which RP and pISD modeling was performed. The final ‘base GRN’ for CellOracle modeling was constructed as the union of the MIRA-derived TF-gene linkages and Cicero-derived TF-gene linkages so that TFs which lack appropriate ChIP-seq data are not removed or penalized in the downstream predictive modeling steps. CellOracle GRN modeling was performed separately for each of the Leiden clusters in the Donor 1 and Donor 2 multiome datasets. After initially performing fitting using all TF-gene links in base-GRN, the top 10,000 TF-gene linkages (p < 0.001) in each cell state were selected for a second round of fitting using Bayesian ridge regression. The resulting second fitted TF-gene linkages were used for final state-specific GRN assembly, centrality inferences, and *in silico* TF perturbation simulations.

### In silico TF perturbation analysis

For *in silico* TF perturbation analysis, differentiation trajectories as inferred by STREAM psuedotime and CellOracle gradient flow (see ‘STREAM psuedotime and differentiation trajectory inference’) were analyzed along two distinct trajectories: PB (early-PB and PB clusters) and GCBCs (GC-1 and GC-2 clusters). For each TF perturbation, the perturbation scores for cells in either PB or GC clusters was computed by calculating the dot production between the reference differentiation flow and the simulated TF perturbation flow. Net perturbation scores for each trajectory were calculated by summing the positive and negative perturbation scores for cells in PB or GC clusters, separately. Then, the overall GC-PB perturbation score was derived by subtracting the net PB perturbation score from the net GC perturbation score for each TF. As with the GRN modeling, this process was conducted separately for male and female datasets.

### Arrayed CRIPSR/Cas9 phenotypic screen

Naïve B cells (NBCs) from either Donor 1 or Donor 2 were isolated and activated as described above. After two days of activation, cells were nucleofected with Cas9:sgRNA ribonucleoprotein (RNP) complexes using a 4D-Nucleofector System (Lonza). To generate RNP complexes, 60 picomoles of arrayed sgRNAs (three per gene/reaction, see Table S13 for sequences) (Synthego) were incubated with 20 picomoles of Cas9 (IDT) (diluted in P3 nucleofection Solution with supplement (Lonza)) each for up to an hour at room temperature. Prior to nucleofection cells were washed with PBS and centrifuged at 90xg for 10 minutes before resuspending in P3 Nucleofection Solution with supplement. 200,000–300,000 cells per reaction were nucleofected in Nucleocuvette streps and re-cultured in activation cocktail as described above. At Day 4, 50,000 cells from each sample were split off for DNA extraction with 50μL of Quick Extract Solution (BioSearch Technologies). Cells were lysed at 68C for 15 minutes followed by 95C for 10 minutes. Primers for flanking the most distal pair of sgRNAs for each gene were used to detect deletions by PCR. At Day 6, cells were harvested for flow cytometry analysis processing as described above. See Table S12 for antibodies used.

### ChromBPNet data analysis

Aggregated cell state-specific ATAC-sequencing reads were extracted from single-cell multiome dataset by using Sinto’s ‘filterbarcodes’ function. Resulting BAM files were used for peak calling with MACS2 ‘callpeak’ function with following options: --shift −75 --ext 150 --pvalue 0.01 --nomodel -B --SPMR. Peaks for each cell cluster were merged to generate a union set of ATAC peaks which were used to train ChromBPNet models for each cell cluster. A Tn5 bias model was trained by running ChromBPNet ‘bias pipeline’ with nonpeak sequences from inaccessible chromatin regions. Bias-corrected BigWig files were generated by running ChromBPNet ‘pred_bw’ and base-pair resolution contribution score predictions were generated by running ChromBPNet ‘contribs_bw’ with the union set of ATAC peaks. The resulting bigWig files were converted to bedGraph files using bigWigToBedGraph (uscs_utilities). Profile contribution score tracks (i.e. predicted contribution to peak profile/shape) were used for all downstream analysis of ChromBPNet models. For accessibility topic TFModisco analysis, ‘contribs_bw’ was also run with the top 10,000 peaks for each accessibility topic. The resulting profile scores AnnData objects were used as input into TFModisco ‘motifs’ function using sampling of 10,000 seqlets and the ‘report’ function using the Jaspar 2024 Core Vertebrates (non-redundant) PFM collection. Seqlets matching motifs in the same Jaspar motif cluster family were merged for quantification of TF motif family seqlets instances across accessibility topics. Homer or CESeek were used to scan for specific single motif instances or composite element motif instances across the union set of peaks or accessibility topic peaks. Aggerated histogram and heatmap plots were generated using ‘plotHeatmap’ function from deepTools.

### CRISPR/Cas9 perturbation with single-cell RNA-sequencing procedure

Naïve B cells from Donor 1 were isolated and activated as described above. After two days of activation, cells were nucleofected with Cas9:sgRNA ribonucleoprotein RNP complexes as described above. After nucleofection, cells were re-cultured in activation media for two days. On Day 4, 50,000 cells per sample were separated for PCR genotyping as described above and 200,000 cells per sample were frozen with Bambanker medium and stored at −80C. An additional 200,000 cells per sample were re-cultured in differentiation media for two days. On Day 6, dead cells were removed by magnetic cell isolation (EasySep Dead Cell Removal (Annexin V) Kit, StemCell Technologies). Live cells were incubated in Human TruStain FcX (Biolegend) for 10 minutes on ice before washing and resuspending in FACS buffer. Cells were then stained with TotalSeq-C anti-human hashtag oligonucleotide conjugated antibodies (Biolegend, see Table S12 for antibody details) for 30 minutes on ice. Cells were then washed two times with 2% FBS/PBS and one time with 1% BSA/PBS before processing with Single-cell 5’ Kit v2 (10x Genomics) and Human BCR Amplification Kit (10x Genomics) according to manufacturer instructions.

### CRISPR/Cas9 perturbation with single-cell RNA-sequencing data analysis

FASTQ files were generated using CellRanger (v7.1.0) ‘mkfastq’ function and used as input into CellRanger ‘multi’ function using GRCh38 (2024) as the reference genome. CellRanger demultiplexed sample feature barcode matrices were merged and MIRA (v2.1.0) expression topic modeling was performed using the most highly variable genes. Following Leiden clustering, differentially expressed genes between KO cells and NTC cells within the Day 4 ActB cluster were identified by using SCANPY’s ‘rank_genes_groups’ function.

### Multiplexed immunofluorescence imaging and analysis

Poly-D-Lysine (Gibco; A3890401) coating was applied to a glass bottom 96 well plate (Cellvis; P96–1.5H-N) for 30 minutes, followed by a phosphate-buffered saline (PBS) wash. Cells were then fixed using paraformaldehyde (Thermo Scientific; 28908) for 15 minutes at room temperature. Permeabilization was carried out with 0.3% Triton X-100(Sigma; T8787) in PBS for 20 minutes, after which blocking was performed for 1 hour at room temperature in a solution containing 10% (w/v) donkey serum (Jackson Immuno Research Labs; NC9624464) and 3% (w/v) bovine serum albumin (Sigma Aldrich; 05470) in PBS. Primary antibodies (see Table S12) were diluted in the blocking buffer and incubated for 1 hour at room temperature. Fluorescently labeled primary antibodies were generated in-house using Alexa Fluor labeling kits: Alexa Fluor 555 (Invitrogen; A20187), Alexa Fluor 647 (Invitrogen; A20186), and Alexa Fluor 750 (Invitrogen; A37575). Nuclear staining was performed using Hoechst (2 μg/mL, Invitrogen; H3570) for 10 minutes at room temperature. Imaging was carried out in a solution of 50% glycerol in PBS. Following image acquisition, fluorescent dyes were quenched using an alkaline hydrogen peroxide solution for 15 minutes under agitation, followed by a PBS wash. To assess residual fluorescence, samples were re-imaged before proceeding with additional rounds of antibody staining and imaging. Image acquisition was performed using the Thunder widefield inverted fluorescence microscope (Leica Biosystems) equipped with a HC PL APO 20x/0.80 objective, Lumencor LED8 illuminator and the following filter sets: DAPI: ex: 391/32, dichroic: 415, em: 435/30; AlexaFluor 488: ex: 479/33, dichroic: 500, em: 519/25; AlexaFluor 555: ex: 554/24, dichroic: 572, em: 594/32; AlexaFluor 647: ex: 638/31, em: 695/58; AlexaFluor 750, ex: 730/40, em: 810/80. Cell segmentation was conducted using Cellpose^83^, while intensity values were extracted via scikit-image^84^. Feature selection was applied as previously established^69^, and cell cycle maps were generated using PHATE^85^, following established methodologies^53^.

## Supplementary Material

Supplement 1

Table S1. Single-cell RNA-seq Leiden cluster marker gene lists.

Table S2. Single-cell multiome expression topic gene lists.

Table S3. Gene ontology analysis of Donor 1 multiome expression topic genes.

Table S4. ChIP-seq peak enrichment of Donor 1 multiome accessibility topic peaks.

Table S5. B cell state specific GRN TF to gene edges.

Table S6. B cell state specific GRN TF centrality scores.

Table S7. ChromBPNet TF motif family seqlet counts per accessibility topic.

Table S8. Normalized TF gene expression across B cell states.

Table S9. RP model scores of seqlet-containing CREs linked to TF target genes.

Table S10. Target genes common to IRF4 and its binding partners.

Table S11. Gene ontology analysis of target genes common to IRF4 and its binding partners.

Table S12. Antibodies used in this study.

## References

[1] Davidson E.H. (2010). Emerging properties of animal gene regulatory networks. Nature 468, 911–920. 10.1038/nature09645. 21164479 PMC3967874

[2] Abadie K., Pease N.A., Wither M.J., and Kueh H.Y. (2019). Order by chance: origins and benefits of stochasticity in immune cell fate control. Current Opinion in Systems Biology 18, 95–103. 10.1016/j.coisb.2019.10.013. 33791444 PMC8009491

[3] Sciammas R., Li Y., Warmflash A., Song Y., Dinner A.R., and Singh H. (2011). An incoherent regulatory network architecture that orchestrates B cell diversification in response to antigen signaling. Molecular Systems Biology 7, 495. 10.1038/msb.2011.25. 21613984 PMC3130558

[4] Laslo P., Spooner C.J., Warmflash A., Lancki D.W., Lee H.-J., Sciammas R., Gantner B.N., Dinner A.R., and Singh H. (2006). Multilineage Transcriptional Priming and Determination of Alternate Hematopoietic Cell Fates. Cell 126, 755–766. 10.1016/j.cell.2006.06.052. 16923394

[5] Graf T., and Enver T. (2009). Forcing cells to change lineages. Nature 462, 587–594. 10.1038/nature08533. 19956253

[6] Preissl S., Gaulton K.J., and Ren B. (2023). Characterizing cis-regulatory elements using single-cell epigenomics. Nat Rev Genet 24, 21–43. 10.1038/s41576-022-00509-1. 35840754 PMC9771884

[7] Badia-i-Mompel P., Wessels L., Müller-Dott S., Trimbour R., Ramirez Flores R.O., Argelaguet R., and Saez-Rodriguez J. (2023). Gene regulatory network inference in the era of single-cell multi-omics. Nat Rev Genet 24, 739–754. 10.1038/s41576-023-00618-5. 37365273

[8] Ellebedy A.H., Jackson K.J.L., Kissick H.T., Nakaya H.I., Davis C.W., Roskin K.M., McElroy A.K., Oshansky C.M., Elbein R., Thomas S., (2016). Defining antigen-specific plasmablast and memory B cell subsets in human blood after viral infection or vaccination. Nat Immunol 17, 1226–1234. 10.1038/ni.3533. 27525369 PMC5054979

[9] Kim W., Zhou J.Q., Horvath S.C., Schmitz A.J., Sturtz A.J., Lei T., Liu Z., Kalaidina E., Thapa M., Alsoussi W.B., (2022). Germinal centre-driven maturation of B cell response to mRNA vaccination. Nature 604, 141–145. 10.1038/s41586-022-04527-1. 35168246 PMC9204750

[10] Victora G.D., and Nussenzweig M.C. (2022). Germinal Centers. Annual Review of Immunology 40, 413–442. 10.1146/annurev-immunol-120419-022408. 35113731

[11] Xu H., Chaudhri V.K., Wu Z., Biliouris K., Dienger-Stambaugh K., Rochman Y., and Singh H. (2015). Regulation of bifurcating B cell trajectories by mutual antagonism between transcription factors IRF4 and IRF8. Nat Immunol 16, 1274–1281. 10.1038/ni.3287. 26437243

[12] Song S., and Matthias P.D. (2018). The Transcriptional Regulation of Germinal Center Formation. Frontiers in Immunology 9. 10.3389/fimmu.2018.02026 PMC6134015 30233601

[13] Ochiai K., Maienschein-Cline M., Simonetti G., Chen J., Rosenthal R., Brink R., Chong A.S., Klein U., Dinner A.R., Singh H., (2013). Transcriptional Regulation of Germinal Center B and Plasma Cell Fates by Dynamical Control of IRF4. Immunity 38, 918–929. 10.1016/j.immuni.2013.04.009. 23684984 PMC3690549

[14] Luo Y., Hitz B.C., Gabdank I., Hilton J.A., Kagda M.S., Lam B., Myers Z., Sud P., Jou J., Lin K., (2020). New developments on the Encyclopedia of DNA Elements (ENCODE) data portal. Nucleic Acids Research 48, D882–D889. 10.1093/nar/gkz1062. 31713622 PMC7061942

[15] Liu C., Martins A.J., Lau W.W., Rachmaninoff N., Chen J., Imberti L., Mostaghimi D., Fink D.L., Burbelo P.D., Dobbs K., (2021). Time-resolved Systems Immunology Reveals a Late Juncture Linked to Fatal COVID-19. Cell 0. 10.1016/j.cell.2021.02.018. PMC7874909 33713619

[16] Taylor J.J., Pape K.A., Steach H.R., and Jenkins M.K. (2015). Apoptosis and antigen affinity limit effector cell differentiation of a single naïve B cell. Science 347, 784–787. 10.1126/science.aaa1342. 25636798 PMC4412594

[17] Duffy K.R., Wellard C.J., Markham J.F., Zhou J.H.S., Holmberg R., Hawkins E.D., Hasbold J., Dowling M.R., and Hodgkin P.D. (2012). Activation-Induced B Cell Fates Are Selected by Intracellular Stochastic Competition. Science 335, 338–341. 22223740 10.1126/science.1213230

[18] Zhou J.H.S., Markham J.F., Duffy K.R., and Hodgkin P.D. (2018). Stochastically Timed Competition Between Division and Differentiation Fates Regulates the Transition From B Lymphoblast to Plasma Cell. Frontiers in Immunology 9. 10.3389/fimmu.2018.02053 PMC6139340 30250473

[19] Jelinek D.F., and Lipsky P.E. (1983). The role of B cell proliferation in the generation of immunoglobulin-secreting cells in man. The Journal of Immunology 130, 2597–2604. 10.4049/jimmunol.130.6.2597. 6189896

[20] Hodgkin P.D., Lee J.H., and Lyons A.B. (1996). B cell differentiation and isotype switching is related to division cycle number. J Exp Med 184, 277–281. 10.1084/jem.184.1.277. 8691143 PMC2192686

[21] Scharer C.D., Barwick B.G., Guo M., Bally A.P.R., and Boss J.M. (2018). Plasma cell differentiation is controlled by multiple cell division-coupled epigenetic programs. Nat Commun 9, 1698. 10.1038/s41467-018-04125-8. 29703886 PMC5923265

[22] Elsner R.A., Smita S., and Shlomchik M.J. (2024). IL-12 induces a B cell-intrinsic IL-12/IFNγ feed-forward loop promoting extrafollicular B cell responses. Nat Immunol 25, 1283–1295. 10.1038/s41590-024-01858-1. 38862796 PMC11992614

[23] Heinzel S., Marchingo J.M., Horton M.B., and Hodgkin P.D. (2018). The regulation of lymphocyte activation and proliferation. Current Opinion in Immunology 51, 32–38. 10.1016/j.coi.2018.01.002. 29414529

[24] Chan W.F., Coughlan H.D., Zhou J.H.S., Keenan C.R., Bediaga N.G., Hodgkin P.D., Smyth G.K., Johanson T.M., and Allan R.S. (2021). Pre-mitotic genome re-organisation bookends the B cell differentiation process. Nat Commun 12, 1344. 10.1038/s41467-021-21536-2. 33637722 PMC7910489

[25] Caron G., Hussein M., Kulis M., Delaloy C., Chatonnet F., Pignarre A., Avner S., Lemarié M., Mahé E.A., Verdaguer-Dot N., (2015). Cell-Cycle-Dependent Reconfiguration of the DNA Methylome during Terminal Differentiation of Human B Cells into Plasma Cells. Cell Reports 13, 1059–1071. 10.1016/j.celrep.2015.09.051. 26565917

[26] Pignarre A., Chatonnet F., Caron G., Haas M., Desmots F., and Fest T. (2021). Plasmablasts derive from CD23– activated B cells after the extinction of IL-4/STAT6 signaling and IRF4 induction. Blood 137, 1166–1180. 10.1182/blood.2020005083. 33150420

[27] Emmett A.M.L., Saadi A., Care M.A., Doody G.M., Tooze R.M., and Westhead D.R. (2023). Integration of chromatin accessibility and gene expression data with cisREAD reveals a switch from PU.1/SPIB-driven to AP-1-driven gene regulation during B cell activation (Bioinformatics) 10.1101/2023.01.09.522862.

[28] Victora G.D., Dominguez-Sola D., Holmes A.B., Deroubaix S., Dalla-Favera R., and Nussenzweig M.C. (2012). Identification of human germinal center light and dark zone cells and their relationship to human B-cell lymphomas. Blood 120, 2240–2248. 10.1182/blood-2012-03-415380. 22740445 PMC3447782

[29] Elgueta R., Benson M.J., de Vries V.C., Wasiuk A., Guo Y., and Noelle R.J. (2009). Molecular mechanism and function of CD40/CD40L engagement in the immune system. Immunol Rev 229, 10.1111/j.1600-065X.2009.00782.x. 10.1111/j.1600-065X.2009.00782.x. PMC3826168 19426221

[30] Lynch A.W., Theodoris C.V., Long H.W., Brown M., Liu X.S., and Meyer C.A. (2022). MIRA: joint regulatory modeling of multimodal expression and chromatin accessibility in single cells. Nat Methods 19, 1097–1108. 10.1038/s41592-022-01595-z. 36068320 PMC9517733

[31] Massoni-Badosa R., Aguilar-Fernández S., Nieto J.C., Soler-Vila P., Elosua-Bayes M., Marchese D., Kulis M., Vilas-Zornoza A., Bühler M.M., Rashmi S., (2024). An atlas of cells in the human tonsil. Immunity 57, 379–399.e18. 10.1016/j.immuni.2024.01.006. 38301653 PMC10869140

[32] Pliner H.A., Packer J.S., McFaline-Figueroa J.L., Cusanovich D.A., Daza R.M., Aghamirzaie D., Srivatsan S., Qiu X., Jackson D., Minkina A., (2018). Cicero Predicts cis-Regulatory DNA Interactions from Single-Cell Chromatin Accessibility Data. Molecular Cell 71, 858–871.e8. 10.1016/j.molcel.2018.06.044. 30078726 PMC6582963

[33] Kamimoto K., Stringa B., Hoffmann C.M., Jindal K., Solnica-Krezel L., and Morris S.A. (2023). Dissecting cell identity via network inference and in silico gene perturbation. Nature 614, 742–751. 10.1038/s41586-022-05688-9. 36755098 PMC9946838

[34] Gschwind A.R., Mualim K.S., Karbalayghareh A., Sheth M.U., Dey K.K., Jagoda E., Nurtdinov R.N., Xi W., Tan A.S., Jones H., (2023). An encyclopedia of enhancer-gene regulatory interactions in the human genome. bioRxiv, 2023.11.09.563812. 10.1101/2023.11.09.563812.

[35] Wang S., Sun H., Ma J., Zang C., Wang C., Wang J., Tang Q., Meyer C.A., Zhang Y., and Liu X.S. (2013). Target analysis by integration of transcriptome and ChIP-seq data with BETA. Nat Protoc 8, 2502–2515. 10.1038/nprot.2013.150. 24263090 PMC4135175

[36] Qin Q., Fan J., Zheng R., Wan C., Mei S., Wu Q., Sun H., Brown M., Zhang J., Meyer C.A., (2020). Lisa: inferring transcriptional regulators through integrative modeling of public chromatin accessibility and ChIP-seq data. Genome Biology 21, 32. 10.1186/s13059-020-1934-6. 32033573 PMC7007693

[37] Wei X., Xiang Y., Peters D.T., Marius C., Sun T., Shan R., Ou J., Lin X., Yue F., Li W., (2022). HiCAR is a robust and sensitive method to analyze open-chromatin-associated genome organization. Molecular Cell 82, 1225–1238.e6. 10.1016/j.molcel.2022.01.023. 35196517 PMC8934281

[38] Cao Z., Sun X., Icli B., Wara A.K., and Feinberg M.W. (2010). Role of Krüppel-like factors in leukocyte development, function, and disease. Blood 116, 4404–4414. 10.1182/blood-2010-05-285353. 20616217 PMC2996110

[39] Dengler H.S., Baracho G.V., Omori S.A., Bruckner S., Arden K., Castrillon D.H., DePinho R.A., and Rickert R.C. (2008). Distinct roles for Foxo1 at multiple stages of B cell differentiation. Nat Immunol 9, 1388–1398. 10.1038/ni.1667. 18978794 PMC2679692

[40] Tang T.F., Chan Y.T., Cheong H.C., Cheok Y.Y., Anuar N.A., Looi C.Y., Gan G.G., and Wong W.F. (2022). Regulatory network of BLIMP1, IRF4, and XBP1 triad in plasmacytic differentiation and multiple myeloma pathogenesis. Cellular Immunology 380, 104594. 10.1016/j.cellimm.2022.104594. 36081178

[41] Hagn M., Sontheimer K., Dahlke K., Brueggemann S., Kaltenmeier C., Beyer T., Hofmann S., Lunov O., Barth T.F.E., Fabricius D., (2012). Human B cells differentiate into granzyme B-secreting cytotoxic B lymphocytes upon incomplete T-cell help. Immunol Cell Biol 90, 457–467. 10.1038/icb.2011.64. 21808264

[42] Chen H., Albergante L., Hsu J.Y., Lareau C.A., Lo Bosco G., Guan J., Zhou S., Gorban A.N., Bauer D.E., Aryee M.J., (2019). Single-cell trajectories reconstruction, exploration and mapping of omics data with STREAM. Nat Commun 10, 1903. 10.1038/s41467-019-09670-4. 31015418 PMC6478907

[43] Laidlaw B.J., and Cyster J.G. (2021). Transcriptional regulation of memory B cell differentiation. Nat Rev Immunol 21, 209–220. 10.1038/s41577-020-00446-2. 33024284 PMC7538181

[44] Glasmacher E., Agrawal S., Chang A.B., Murphy T.L., Zeng W., Vander Lugt B., Khan A.A., Ciofani M., Spooner C.J., Rutz S., (2012). A genomic regulatory element that directs assembly and function of immune-specific AP-1-IRF complexes. Science 338, 975–980. 10.1126/science.1228309. 22983707 PMC5789805

[45] Brass A.L., Zhu A.Q., and Singh H. (1999). Assembly requirements of PU.1–Pip (IRF-4) activator complexes: inhibiting function in vivo using fused dimers. EMBO J 18, 977–991. 10.1093/emboj/18.4.977. 10022840 PMC1171190

[46] Minnich M., Tagoh H., Bönelt P., Axelsson E., Fischer M., Cebolla B., Tarakhovsky A., Nutt S.L., Jaritz M., and Busslinger M. (2016). Multifunctional role of the transcription factor Blimp-1 in coordinating plasma cell differentiation. nature immunology 17. 10.1038/ni.3349 PMC5790184 26779602

[47] Pampari A., Shcherbina A., Nair S., Schreiber J., Patel A., Wang A., Kundu S., Shrikumar A., and Kundaje A. (2023). Bias factorized, base-resolution deep learning models of chromatin accessibility reveal cis-regulatory sequence syntax, transcription factor footprints and regulatory variants. Version 0.1.1. 10.5281/zenodo.7567627 https://doi.org/10.5281/zenodo.7567627.

[48] Pampari A., Shcherbina A., Kvon E.Z., Kosicki M., Nair S., Kundu S., Kathiria A.S., Risca V.I., Kuningas K., Alasoo K., (2024). ChromBPNet: bias factorized, base-resolution deep learning models of chromatin accessibility reveal cis-regulatory sequence syntax, transcription factor footprints and regulatory variants. Preprint, 10.1101/2024.12.25.630221 https://doi.org/10.1101/2024.12.25.630221.

[49] Shrikumar A., Tian K., Avsec Ž., Shcherbina A., Banerjee A., Sharmin M., Nair S., and Kundaje A. (2020). Technical Note on Transcription Factor Motif Discovery from Importance Scores (TF-MoDISco) version 0.5.6.5. Preprint at arXiv.

[50] Chaudhri V.K., and Singh H. (2021). Diverse digital and fuzzy composite transcriptional elements are prevalent features of mammalian cis-regulomes (Genomics) 10.1101/2021.11.26.470154.

[51] García-Gutiérrez L., Bretones G., Molina E., Arechaga I., Symonds C., Acosta J.C., Blanco R., Fernández A., Alonso L., Sicinski P., (2019). Myc stimulates cell cycle progression through the activation of Cdk1 and phosphorylation of p27. Sci Rep 9, 18693. 10.1038/s41598-019-54917-1. 31822694 PMC6904551

[52] Susaki E., Nakayama K., and Nakayama K.I. (2007). Cyclin D2 Translocates p27 out of the Nucleus and Promotes Its Degradation at the G0-G1 Transition. Mol Cell Biol 27, 4626–4640. 10.1128/MCB.00862-06. 17452458 PMC1951473

[53] Stallaert W., Kedziora K.M., Taylor C.D., Zikry T.M., Ranek J.S., Sobon H.K., Taylor S.R., Young C.L., Cook J.G., and Purvis J.E. (2022). The structure of the human cell cycle. Cell Systems 13, 230–240.e3. 10.1016/j.cels.2021.10.007. 34800361 PMC8930470

[54] Kueh H.Y., Champhekhar A., Nutt S.L., Elowitz M.B., and Rothenberg E.V. (2013). Positive feedback between PU.1 and the cell cycle controls myeloid differentiation. Science 341, 670–673. 10.1126/science.1240831. 23868921 PMC3913367

[55] DeKoter R.P., and Singh H. (2000). Regulation of B Lymphocyte and Macrophage Development by Graded Expression of PU.1. Science 288, 1439–1441. 10.1126/science.288.5470.1439. 10827957

[56] Lu X., Chen X., Forney C., Donmez O., Miller D., Parameswaran S., Hong T., Huang Y., Pujato M., Cazares T., (2021). Global discovery of lupus genetic risk variant allelic enhancer activity. Nat Commun 12, 1611. 10.1038/s41467-021-21854-5. 33712590 PMC7955039

[57] Li C., Gao H., She Y., Bian H., Chen Q., Liu K., Wei L., and Zhang X. (2024). Benchmarking AI Models for In Silico Gene Perturbation of Cells. Preprint, 10.1101/2024.12.20.629581 https://doi.org/10.1101/2024.12.20.629581.

[58] Engreitz J.M., Lawson H.A., Singh H., Starita L.M., Hon G.C., Carter H., Sahni N., Reddy T.E., Lin X., Li Y., (2024). Deciphering the impact of genomic variation on function. Nature 633, 47–57. 10.1038/s41586-024-07510-0. 39232149 PMC11973978

[59] Mittrücker H.W., Matsuyama T., Grossman A., Kündig T.M., Potter J., Shahinian A., Wakeham A., Patterson B., Ohashi P.S., and Mak T.W. (1997). Requirement for the transcription factor LSIRF/IRF4 for mature B and T lymphocyte function. Science 275, 540–543. 10.1126/science.275.5299.540. 8999800

[60] Sciammas R., Shaffer A.L., Schatz J.H., Zhao H., Staudt L.M., and Singh H. (2006). Graded Expression of Interferon Regulatory Factor-4 Coordinates Isotype Switching with Plasma Cell Differentiation. Immunity 25, 225–236. 10.1016/j.immuni.2006.07.009. 16919487

[61] Shapiro-Shelef M., Lin K.-I., McHeyzer-Williams L.J., Liao J., McHeyzer-Williams M.G., and Calame K. (2003). Blimp-1 is required for the formation of immunoglobulin secreting plasma cells and pre-plasma memory B cells. Immunity 19, 607–620. 10.1016/s1074-7613(03)00267-x. 14563324

[62] Zhang T., Gonzalez D.G., Cote C.M., Kerfoot S.M., Deng S., Cheng Y., Magari M., and Haberman A.M. (2017). Germinal center B cell development has distinctly regulated stages completed by disengagement from T cell help. eLife 6, e19552. 10.7554/eLife.19552. PMC5429091 28498098

[63] Xiong E., Popp O., Salomon C., Mertins P., Kocks C., Rajewsky K., and Chu V.T. (2023). A CRISPR/Cas9-mediated screen identifies determinants of early plasma cell differentiation. Front. Immunol. 13. 10.3389/fimmu.2022.1083119. PMC9849354 36685499

[64] Pinter T., Fischer M., Schäfer M., Fellner M., Jude J., Zuber J., Busslinger M., and Wöhner M. (2022). Comprehensive CRISPR-Cas9 screen identifies factors which are important for plasmablast development. Front. Immunol. 13. 10.3389/fimmu.2022.979606. PMC9521597 36189249

[65] Trezise S., Kong I.Y., Hawkins E.D., Herold M.J., Willis S.N., and Nutt S.L. (2023). An arrayed CRISPR screen of primary B cells reveals the essential elements of the antibody secretion pathway. Front. Immunol. 14. 10.3389/fimmu.2023.1089243. PMC9969136 36860866

[66] Shimshon L., Michaeli A., Hadar R., Nutt S.L., David Y., Navon A., Waisman A., and Tirosh B. (2011). SUMOylation of Blimp-1 promotes its proteasomal degradation. FEBS Letters 585, 2405–2409. 10.1016/j.febslet.2011.06.022. 21722636

[67] Gaudette B.T., Roman C.J., Ochoa T.A., Gómez Atria D., Jones D.D., Siebel C.W., Maillard I., and Allman D. (2021). Resting innate-like B cells leverage sustained Notch2/mTORC1 signaling to achieve rapid and mitosis-independent plasma cell differentiation. Journal of Clinical Investigation 131, e151975. 10.1172/JCI151975. PMC8516456 34473651

[68] Tourigny M.R., Ursini-Siegel J., Lee H., Toellner K.-M., Cunningham A.F., Franklin D.S., Ely S., Chen M., Qin X.-F., Xiong Y., (2002). CDK Inhibitor p18INK4c Is Required for the Generation of Functional Plasma Cells. Immunity 17, 179–189. 10.1016/S1074-7613(02)00364-3. 12196289

[69] Stallaert W., Taylor S.R., Kedziora K.M., Taylor C.D., Sobon H.K., Young C.L., Limas J.C., Varblow Holloway J., Johnson M.S., Cook J.G., (2022). The molecular architecture of cell cycle arrest. Molecular Systems Biology 18, e11087. 10.15252/msb.202211087. PMC9511499 36161508

[70] Spencer S.L., Cappell S.D., Tsai F.-C., Overton K.W., Wang C.L., and Meyer T. (2013). The Proliferation-Quiescence Decision Is Controlled by a Bifurcation in CDK2 Activity at Mitotic Exit. Cell 155, 369–383. 10.1016/j.cell.2013.08.062. 24075009 PMC4001917

[71] Dahl R., Walsh J.C., Lancki D., Laslo P., Iyer S.R., Singh H., and Simon M.C. (2003). Regulation of macrophage and neutrophil cell fates by the PU.1:C/EBPα ratio and granulocyte colony-stimulating factor. Nat Immunol 4, 1029–1036. 10.1038/ni973. 12958595

[72] Vardaka P., Page E., Care M.A., Stephenson S., Kemp B., Umpierrez M., O’Callaghan E., Mabbutt A., Owen R., Hodson D.J., (2024). Enforced MYC expression selectively redirects transcriptional programs during human plasma cell differentiation. bioRxiv, 2024.04.18.589889. 10.1101/2024.04.18.589889.

[73] Gao F.-B., Durand B., and Raff M. (1997). Oligodendrocyte precursor cells count time but not cell divisions before differentiation. Current Biology 7, 152–155. 10.1016/S0960-9822(06)00060-1. 9016704

[74] Haas M., Caron G., Chatonnet F., Manenti S., Alaterre E., Devin J., Delaloy C., Bertolin G., Viel R., Pignarre A., (2022). PIM2 kinase has a pivotal role in plasmablast generation and plasma cell survival, opening up novel treatment options in myeloma. Blood 139, 2316–2337. 10.1182/blood.2021014011. 35108359

[75] Paus D., Phan T.G., Chan T.D., Gardam S., Basten A., and Brink R. (2006). Antigen recognition strength regulates the choice between extrafollicular plasma cell and germinal center B cell differentiation. Journal of Experimental Medicine 203, 1081–1091. 10.1084/jem.20060087. 16606676 PMC2118299

[76] Plambeck M., Kazeroonian A., Loeffler D., Kretschmer L., Salinno C., Schroeder T., Busch D.H., Flossdorf M., and Buchholz V.R. (2022). Heritable changes in division speed accompany the diversification of single T cell fate. Proceedings of the National Academy of Sciences 119, e2116260119. 10.1073/pnas.2116260119. PMC8892279 35217611

[77] Kretschmer L., Flossdorf M., Mir J., Cho Y.-L., Plambeck M., Treise I., Toska A., Heinzel S., Schiemann M., Busch D.H., (2020). Differential expansion of T central memory precursor and effector subsets is regulated by division speed. Nat Commun 11, 113. 10.1038/s41467-019-13788-w. 31913278 PMC6949285

[78] McGinnis C.S., Patterson D.M., Winkler J., Conrad D.N., Hein M.Y., Srivastava V., Hu J.L., Murrow L.M., Weissman J.S., Werb Z., (2019). MULTI-seq: sample multiplexing for single-cell RNA sequencing using lipid-tagged indices. Nat Methods 16, 619–626. 10.1038/s41592-019-0433-8. 31209384 PMC6837808

[79] Wolf F.A., Angerer P., and Theis F.J. (2018). SCANPY: large-scale single-cell gene expression data analysis. Genome Biol 19, 15. 10.1186/s13059-017-1382-0. 29409532 PMC5802054

[80] Chen D., Wang Y., Manakkat Vijay G.K., Fu S., Nash C.W., Xu D., He D., Salomonis N., Singh H., and Xu H. (2021). Coupled analysis of transcriptome and BCR mutations reveals role of OXPHOS in affinity maturation. Nat Immunol, 1–10. 10.1038/s41590-021-00936-y. 34031613

[81] Zheng R., Wan C., Mei S., Qin Q., Wu Q., Sun H., Chen C.-H., Brown M., Zhang X., Meyer C.A., (2019). Cistrome Data Browser: expanded datasets and new tools for gene regulatory analysis. Nucleic Acids Research 47, D729–D735. 10.1093/nar/gky1094. 30462313 PMC6324081

[82] Lambert S.A., Jolma A., Campitelli L.F., Das P.K., Yin Y., Albu M., Chen X., Taipale J., Hughes T.R., and Weirauch M.T. (2018). The Human Transcription Factors. Cell 172, 650–665. 10.1016/j.cell.2018.01.029. 29425488 PMC12908702

[83] Pachitariu M., and Stringer C. (2022). Cellpose 2.0: how to train your own model. Nat Methods 19, 1634–1641. 10.1038/s41592-022-01663-4. 36344832 PMC9718665

[84] van der Walt S., Schönberger J.L., Nunez-Iglesias J., Boulogne F., Warner J.D., Yager N., Gouillart E., Yu T., and contributors, the scikit-image (2014). scikit-image: image processing in Python. PeerJ 2, e453. 10.7717/peerj.453. 25024921 PMC4081273

[85] Moon K.R., van Dijk D., Wang Z., Gigante S., Burkhardt D.B., Chen W.S., Yim K., Elzen A. van den, Hirn M.J., Coifman R.R., (2019). Visualizing structure and transitions in high-dimensional biological data. Nature Biotechnology 37, 1482–1492. 10.1038/s41587-019-0336-3. PMC7073148 31796933

