# Supplementary material for: Cell cycle-coupled transcriptional network orchestrates human B cell fate bifurcation
Source: bioRxiv. 2025 Jun 25:2025.04.23.649973. Preprint. [Version 2] doi: 10.1101/2025.04.23.649973 (PMC12262194; doi:10.1101/2025.04.23.649973)
Supplement: Supplement 1 [file NIHPP2025.04.23.649973v2-supplement-1.pdf]

Supplemental Figure 1

bioRxiv preprint doi: https://doi.org/10.1101/2025.04.25.649973; this version posted June 25, 2025. The copyright holder for this preprint (which was not certified by peer review) is the author/funder, who has granted bioRxiv a license to display the preprint in perpetuity. It is made available under aCC-BY 4.0 International license.

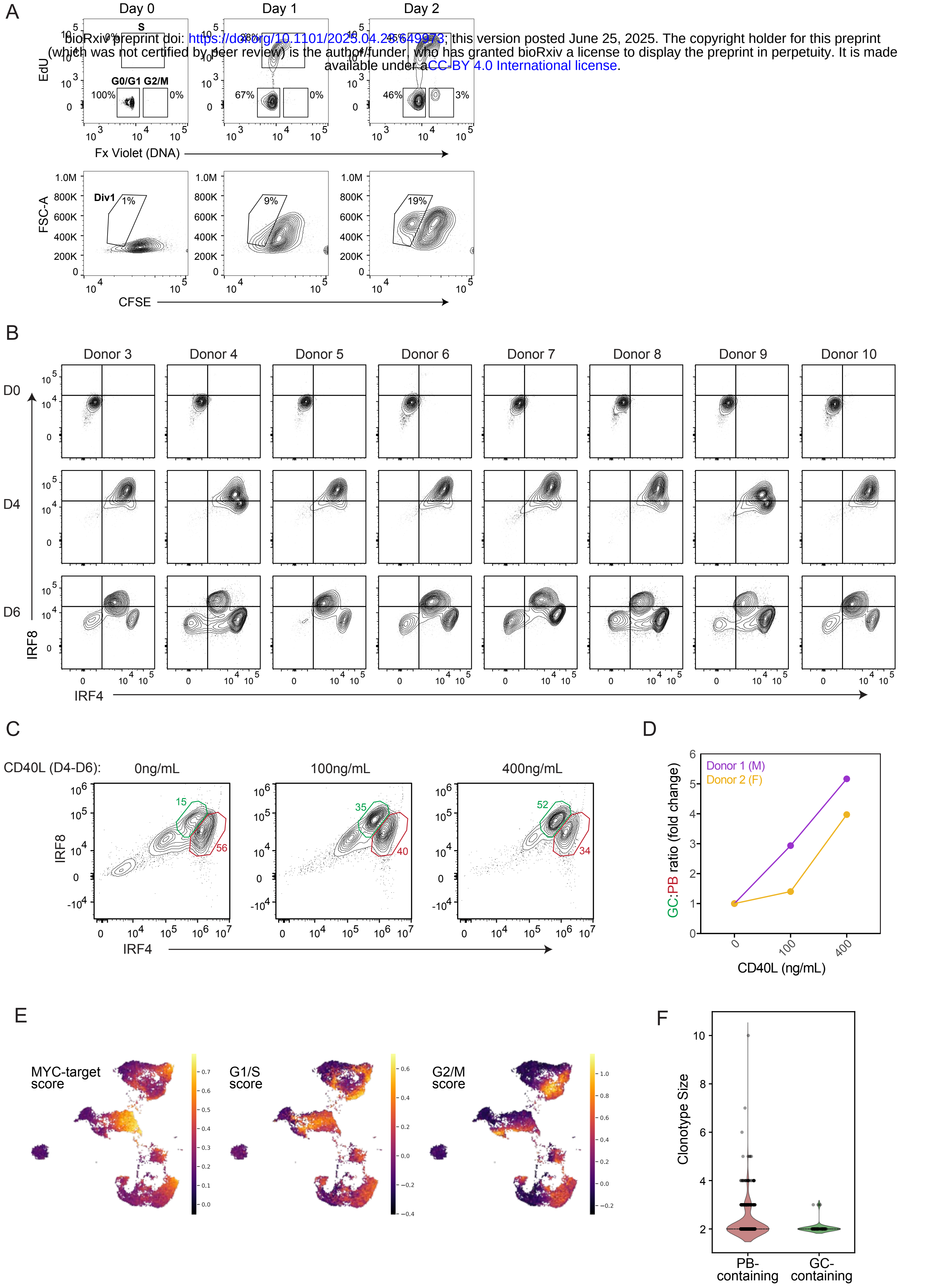

bioRxiv preprint doi: https://doi.org/10.1101/2025.04.23.649973; this version posted June 25, 2025. The copyright holder for this preprint (which was not certified by peer review) is the author/funder, who has granted bioRxiv a license to display the preprint in perpetuity. It is made available under aCC-BY 4.0 International license.

Figure S1. Human B cells activated *in vitro* bifurcate into PB and GC fates. (A) Naïve B cells were labeled with CFSE proliferation dye, stimulated as in Figure 1A and pulsed with EdU thymidine analog for two hours at indicated timepoints to measure *de novo* DNA synthesis before fixing for flow cytometry analysis. (B) Analysis of bifurcating dynamics of IRF4 and IRF8 expression in activated B cells from 8 additional donors (4 male and 4 female) by flow cytometry, as in Figure 1A. (C) Naïve B cells were stimulated for 4 days as in Figure 1A, before re-culturing with indicated concentrations of CD40L in the presence of IL-2, IL-4 and IL-10 for 2 days, and their analysis by flow cytometry at D6. (D) GC:PB ratios (D6) as a function of CD40L concentration are displayed for two donors (male and female). (E) UMAP projections of scRNA-seq data (see Figure 1C) displaying MYC-target and cell-cycle gene scores[80]. (F) Distribution of clonotype sizes for each cell fate category (n = number of unique clonotypes of size >= 2).

Supplemental Figure 2

bioRxiv preprint doi: https://doi.org/10.1101/2025.04.23.649973; this version posted June 25, 2025. The copyright holder for this preprint (which was not certified by peer review) is the author/funder, who has granted bioRxiv a license to display the preprint in perpetuity. It is made available under aCC-BY 4.0 International license.

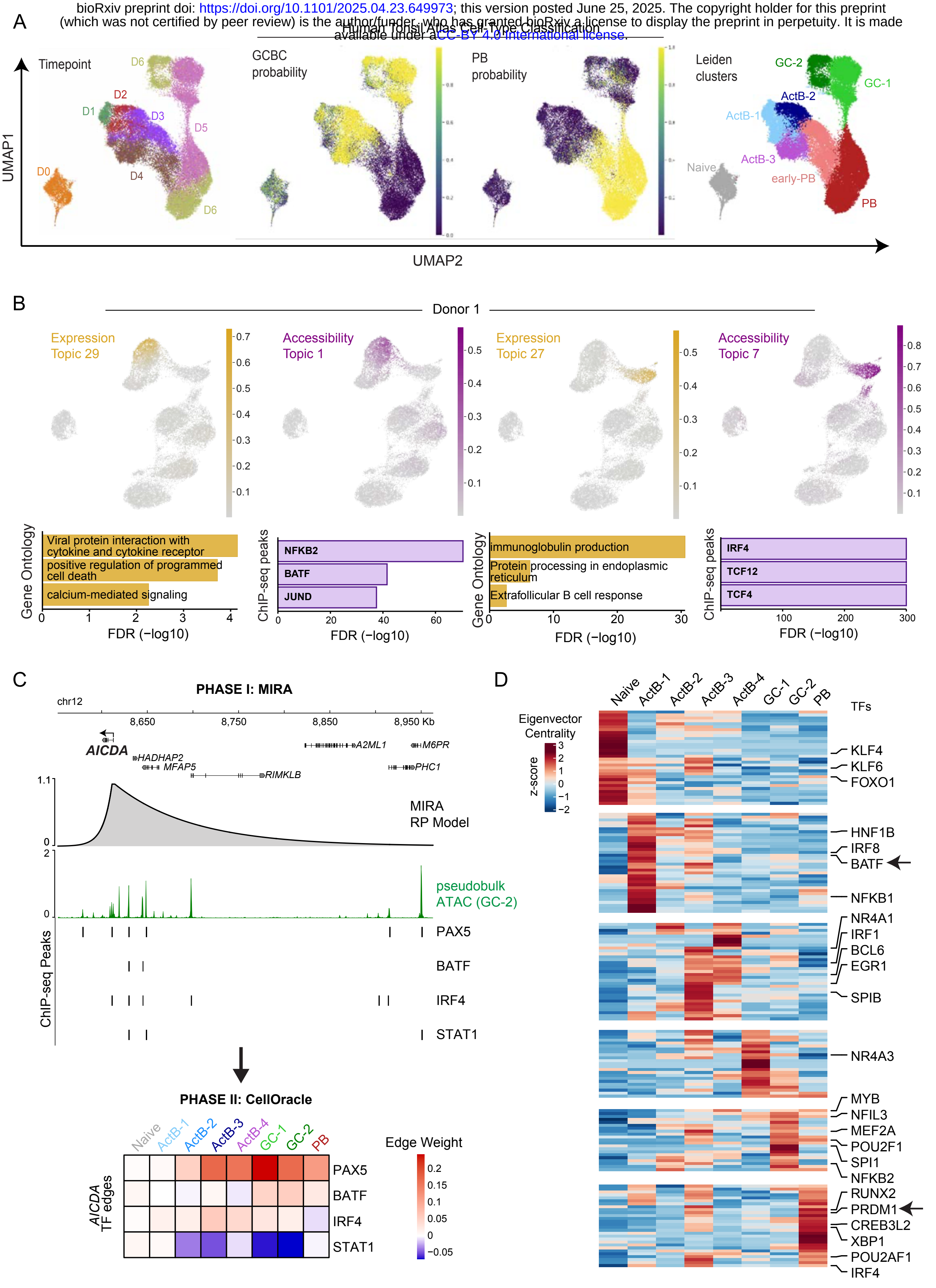

bioRxiv preprint doi: https://doi.org/10.1101/2025.04.23.649973; this version posted June 25, 2025. The copyright holder for this preprint (which was not certified by peer review) is the author/funder, who has granted bioRxiv a license to display the preprint in perpetuity. It is made available under aCC-BY 4.0 International license.

Figure S2. Assembly of GRNs underlying bifurcation of human B cells into PB and GC fates. (A) Naïve human B cells from Donor 2 (female) were activated and differentiated, *in vitro*, as in Figure 1A and profiled every 24 hours by paired single-cell RNA/ATAC-seq. MIRA joint topic modeling of transcripts and chromatin accessibility features[30] was used to project cells in low-dimensional UMAP space and colored based on timepoint (*left*), support vector machine classification probabilities based on Human Tonsil Atlas cell-types (*middle*), and Leiden clusters with their cell-type annotations (*right*). (B) UMAP projections of expression topic composition (gold) and accessibility topic composition (purple) are displayed for representative GC-associated (topic 29 and topic 1) or PB-associated (topic 27 and topic 7) topics. Gene ontology analysis of the top 250 genes is displayed beneath each indicated expression topic and ChIP-seq peak enrichment for the top 10,000 peaks is displayed beneath each indicated accessibility topic. (C) Representative RP model for *AICDA* gene with ATAC-seq signals derived from the GC-2 state pseudobulk shown below. ChIP-seq peaks (CistromeDB) that overlap with ATAC-seq peaks for representative TF regulators predicted by MIRA pISD modeling are shown below ATAC-seq track. Representative CellOracle state-specific TF-gene edge weights for MIRA predicted regulators of *AICDA* are shown below ChIP-seq peak tracks. (D) Heatmap displaying the CellOracle state-specific eigenvector centrality scores for TFs with a score of > 0.005 in at least one cell state. Values are z-scaled across indicated cell states. TFs (rows) were k-means clustered and arranged based on B cell activation and differentiation sequence. Representative TFs are indicated.

Supplemental Figure 3

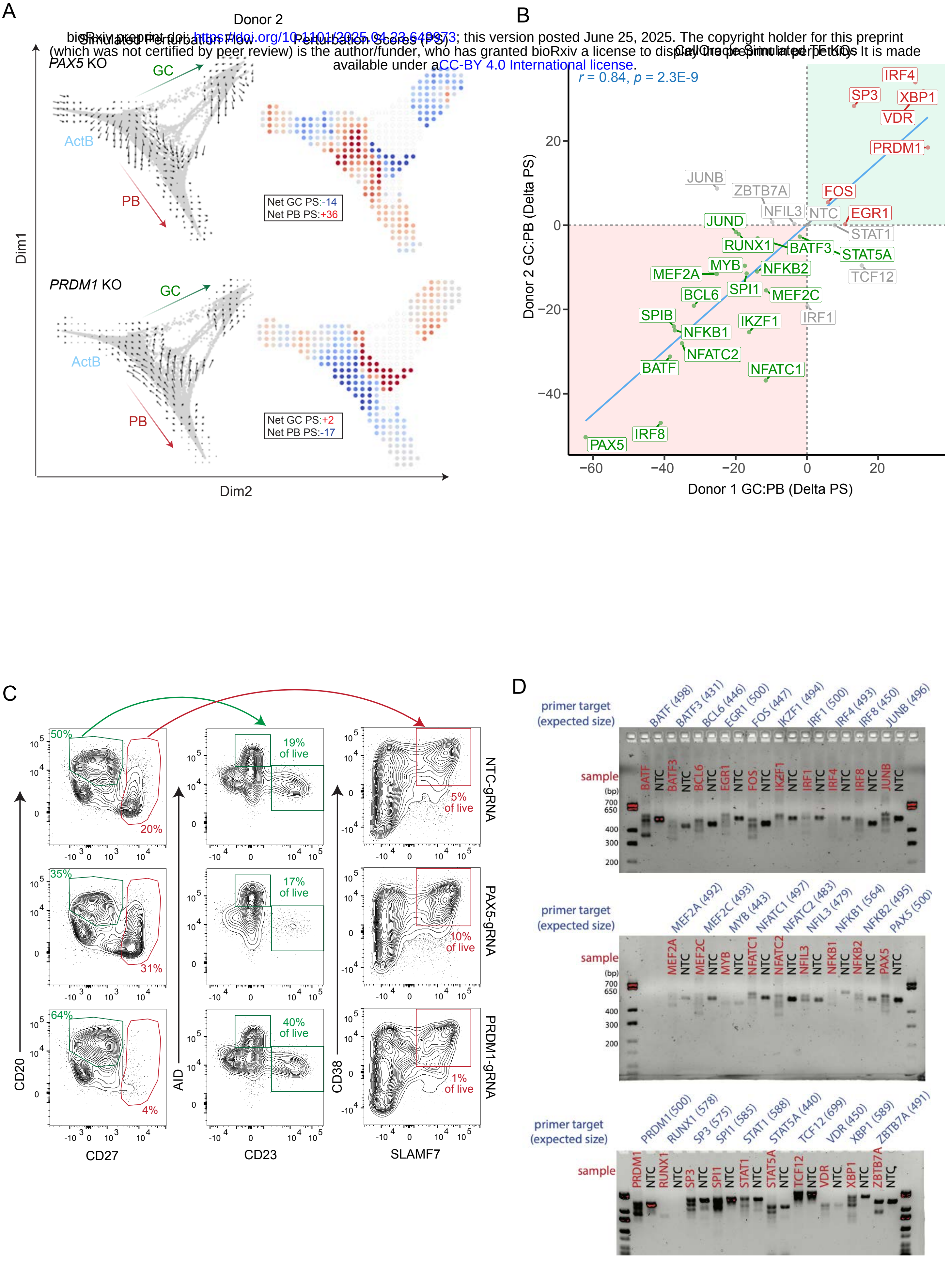

bioRxiv preprint doi: https://doi.org/10.1101/2025.04.22.649973; this version posted June 25, 2025. The copyright holder for this preprint (which was not certified by peer review) is the author/funder, who has granted bioRxiv a license to display the preprint in perpetuity. It is made available under aCC-BY 4.0 International license.

bioRxiv preprint doi: https://doi.org/10.1101/2025.04.23.649973; this version posted June 25, 2025. The copyright holder for this preprint (which was not certified by peer review) is the author/funder, who has granted bioRxiv a license to display the preprint in perpetuity. It is made available under aCC-BY 4.0 International license.

Figure S3. Predicting and testing TF control of human B cell fate choice. (A) Representative *in silico* TF KO simulations visualized with perturbation flow vector fields (*left*) or perturbation score grids (*right*) for PAX5 and PRDM1 (Donor 2, female). Perturbation scores (PS) based on differentiation flow and perturbation simulation vectors were computed as in Figure 3B. (B) Comparison of Donor 1 (male) and Donor 2 (female) CellOracle TF KO simulation predictions, R = Pearson correlation coefficient). TFs with concordant effects (>= 25% changes in GC:PB ratio) between donors are highlighted in red (PB-promoting TF) or green (GC-promoting TF). (C) Representative flow cytometry plots displaying gating strategy for quantifying PB and GC cells at Day 6 (NTC) and the phenotypic impact of *PAX5* or *PRDM1* CRISPR KO. (D) PCR genotyping results confirming CRISPR editing activity at indicated TF genes. Primers flanking the two most distal gRNA target sequences were used to detect large indels.

Supplemental Figure 4

bioRxiv preprint doi: https://doi.org/10.1101/2025.04.23.649973; this version posted June 25, 2025. The copyright holder for this preprint (which was not certified by peer review) is the author/funder, who has granted bioRxiv a license to display the preprint in perpetuity. It is made available under aCC-BY 4.0 International license.

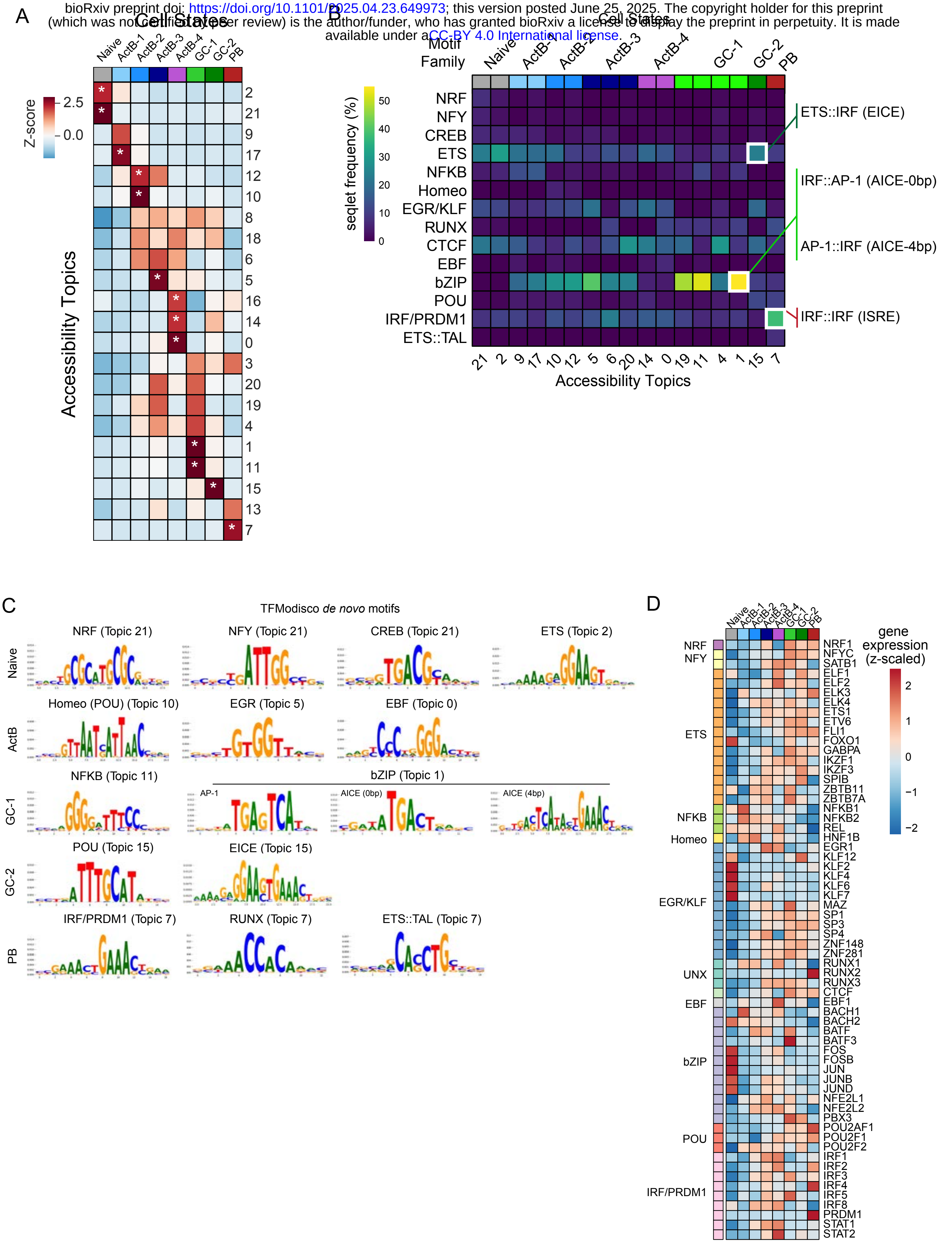

bioRxiv preprint doi: https://doi.org/10.1101/2025.04.23.649973; this version posted June 25, 2025. The copyright holder for this preprint (which was not certified by peer review) is the author/funder, who has granted bioRxiv a license to display the preprint in perpetuity. It is made available under aCC-BY 4.0 International license.

Figure S4. ChromBPNet uncovers dominant and reciprocal TF action at IRF motifs during B cell fate specification. (A) Heatmap displaying z-scores of chromatin accessibility topics in the indicated cell states. Mean composition values of each chromatin topic (rows) in each cell state (columns) were used to generate the heatmap. Asterisks indicate topics associated with a particular cell state with z-scores > 2. (B) Heatmap displaying the TFModisco-derived seqlet frequency for a given TF motif family (related to Figure 4C). Analysis was performed using top 10,000 peaks of indicated state-specific accessibility topics paired with their state-specific ChromBPNet models. Seqlets were annotated using Jaspar 2024 motif cluster families and z-scaled across columns reflective of indicated cell states. Only motif families with a seqlet frequency of at least 5% within at least one topic are displayed (*left*). CWMs for IRF-motif containing elements (EICE, AICE and ISRE) in GC versus PB states are shown (*right*). (C) Representative *de novo* TF motifs identified by TFModisco are displayed for indicated accessibility topics with their associated cell states indicated on the left. (D) Heatmap displaying RNA expression of genes encoding indicated TF family members associated with a given TF motif across various B cell states.

Supplemental Figure S5

bioRxiv preprint doi: https://doi.org/10.1101/2025.04.23.649973; this version posted June 25, 2025. The copyright holder for this preprint (which was not certified by peer review) is the author/funder, who has granted bioRxiv a license to display the preprint in perpetuity. It is made available under aCC-BY 4.0 International license.

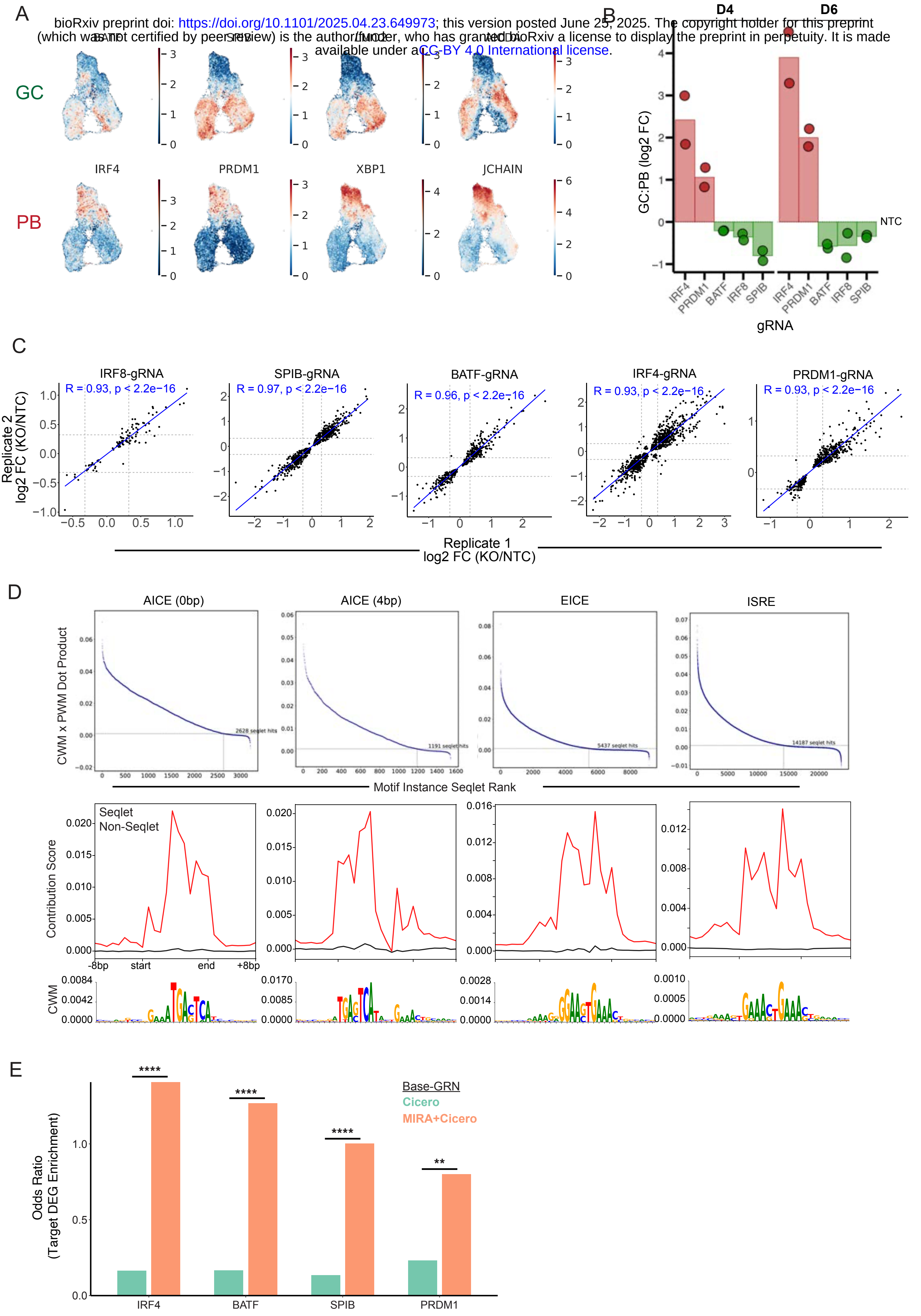

bioRxiv preprint doi: https://doi.org/10.1101/2025.04.23.649973; this version posted June 25, 2025. The copyright holder for this preprint (which was not certified by peer review) is the author/funder, who has granted bioRxiv a license to display the preprint in perpetuity. It is made available under aCC-BY 4.0 International license.

Figure S5. Single-cell perturbation analysis of counteracting PB and GCBC TFs. (A) UMAPs displaying GC and PB marker genes using aggregated scRNA-seq dataset (see Figure 5A). (B) Plot displaying relative proportions of GC and PB cells at Day 6 in each TF-KO condition compared to NTC-gRNA cells (n = 2 experimental replicates using Donor 1 cells). (C) Plots displaying reproducibility between experimental replicates (log2 fold change (FC) in gene expression) for each TF condition (R = Pearson correlation coefficient). Only genes that are statistically significant (FDR < 0.05) in both replicates are shown, irrespective of directionality. Gray lines indicate |FC| of 1.25. (D) Genome-wide ChromBPNet contribution score seqlet-analyses for IRF-containing composite element motifs the ActB-3 cell state. Motif instances are displayed in descending order based on their CWM x PWM dot products (*top*). Dot products were normalized to respective motif lengths. Dotted lines indicate threshold (0.01) set for calling a motif instance as a seqlet hit. Aggregated contribution score signal at seqlet versus non-seqlet motif instances with CWM logo for seqlet motif instances are shown below each set of panels (*bottom*). (E) Fisher's exact test was performed to compare each TF-KO DEG set enrichment within Cicero-only or MIRA+Cicero base-GRN TF-gene linkages (**P<0.01, ****<0.0001).

Supplemental Figure 6

bioRxiv preprint doi: https://doi.org/10.1101/2025.04.23.649973; this version posted June 25, 2025. The copyright holder for this preprint (which was not certified by peer review) is the author/funder, who has granted bioRxiv a license to display the preprint in perpetuity. It is made available under aCC-BY 4.0 International license.

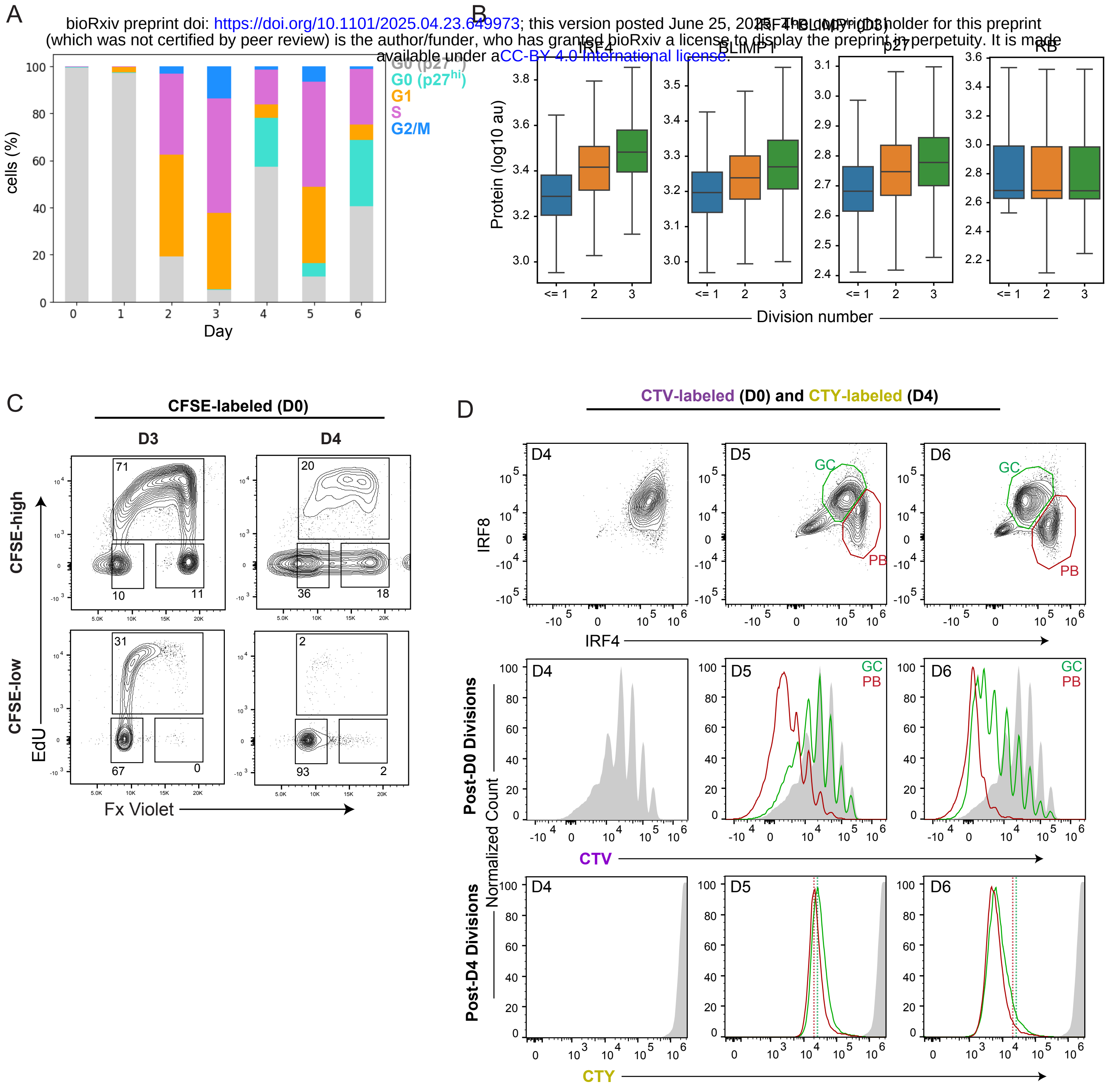

bioRxiv preprint doi: https://doi.org/10.1101/2025.04.23.649973; this version posted June 25, 2025. The copyright holder for this preprint (which was not certified by peer review) is the author/funder, who has granted bioRxiv a license to display the preprint in perpetuity. It is made available under aCC-BY 4.0 International license.

Figure S6. Cell cycle-coupled transcriptional network drives B cell fate choice. (A) Quantification of cell cycle distributions for each timepoint derived from multiplexed imaging cell cycle maps (Figure 6D). (B) Distributions of IRF4, BLIMP1, p27, and RB protein levels as a function of cell division number (derived from CFSE signal) quantified by multiplexed imaging of D3 cells (gated on $IRF4^{lo}BLIMP^{lo}$ cells). (C) Effect of cell division number on cell cycle status. Naïve B cells were labeled with CFSE at D0 and pulsed with EdU for two hours at indicated days before harvesting for flow cytometry analysis (D) Analysis of cell divisions completed by GC or PB cells between D0 and D6 (‘post-D0 divisions’) or between D4 and D6 (‘post-D4 divisions’). Naïve B cells were labeled with CellTrace Violet (CTV, D0), activated for four days as in Figure 1A before harvesting for labeling with CellTrace Yellow (CTY, D4). Cells were then re-cultured in differentiation media for two days. Flow cytometry analyses of IRF4 and IRF8 (*top*), CTV (*middle*) and CTY (*bottom*), performed at D4, D5 and D6, are displayed.
